# Catalysis by design: development of a bifunctional water splitting catalyst through an operando measurement directed optimization cycle†
†Electronic supplementary information (ESI) available. See DOI: 10.1039/c8sc01415a


**DOI:** 10.1039/c8sc01415a

**Published:** 2018-05-08

**Authors:** Nikolay Kornienko, Nina Heidary, Giannantonio Cibin, Erwin Reisner

**Affiliations:** a Department of Chemistry , University of Cambridge , Lensfield Road , Cambridge CB2 1EW , UK . Email: reisner@ch.cam.ac.uk; b Diamond Light Source Ltd. , Diamond House, Harwell Science and Innovation Campus , Didcot OX11 0DE , UK

## Abstract

A critical challenge in energy research is the development of earth abundant and cost-effective materials that catalyze the electrochemical splitting of water into hydrogen and oxygen at high rates and low overpotentials. Key to addressing this issue lies not only in the synthesis of new materials, but also in the elucidation of their active sites, their structure under operating conditions and ultimately, extraction of the structure–function relationships used to spearhead the next generation of catalyst development. In this work, we present a complete cycle of synthesis, operando characterization, and redesign of an amorphous cobalt phosphide (CoP_*x*_) bifunctional catalyst. The research was driven by integrated electrochemical analysis, Raman spectroscopy and gravimetric measurements utilizing a novel quartz crystal microbalance spectroelectrochemical cell to uncover the catalytically active species of amorphous CoP_*x*_ and subsequently modify the material to enhance the activity of the elucidated catalytic phases. Illustrating the power of our approach, the second generation cobalt–iron phosphide (CoFeP_x_) catalyst, developed through an iteration of the operando measurement directed optimization cycle, is superior in both hydrogen and oxygen evolution reactivity over the previous material and is capable of overall water electrolysis at a current density of 10 mA cm^–2^ with 1.5 V applied bias in 1 M KOH electrolyte solution.

## Abstract 2

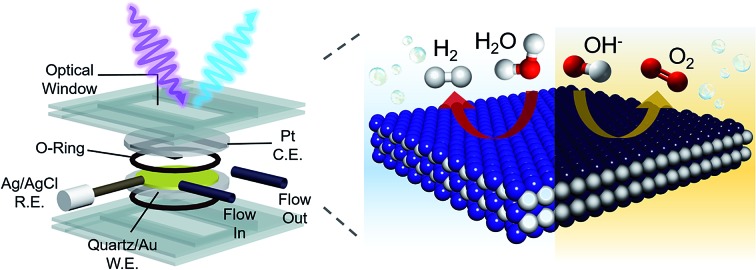
Amorphous and bifunctional water splitting catalysts are probed with a novel spectroelectrochemical quartz crystal microbalance cell, then subsequently enhanced.

## Introduction

The potential use of renewable energy derived hydrogen gas (H_2_) as an energy vector is heavily dependent on the discovery of catalysts that facilitate its generation and subsequent utilization.^1^ In part, scaling H_2_ generation and use to grid-level requires water splitting catalysts that are facile to prepare, are composed of earth-abundant components, and rival the performance of state-of-the-art precious metal based devices.^2^,^3^ The search for such materials spans several decades and from these efforts three primary classes of hydrogen evolution reaction (HER) and oxygen evolution reaction (OER) have emerged: (1) molecular catalysts feature well-defined active sites and exquisite tunability of functional ligands. As such, they are regarded as an ideal platform to explore particular structure–function relationships and to derive guidelines for future catalyst design.^4^,^5^ However, their stability challenges their prospects for practical use. (2) Crystalline catalysts by definition exhibit a well-defined long-range order of their constituent atoms and in certain cases, the arrangement of these atoms on the surface, where catalysis occurs, is precisely known. Their robustness paired with high activity renders them more suitable for practical applications compared to molecular catalysts.^6^ (3) Amorphous catalysts, on the other hand, are commonly defined by what we do not know (crystalline arrangement, composition, active sites…) rather than what we do know about them. However, the activity of amorphous catalysts in many cases surpasses that of their molecular and crystalline analogues.^7^–^9^

In this regard, the lack of rigidity in their atomic order endows a certain adaptability to amorphous materials that allows them to undergo various phase and oxidation changes with changing reaction conditions and facilitate the formation and breakage of chemical bonds, thereby potentially lowering kinetic barriers. Nevertheless, underpinning the development of amorphous catalysts is the need to elucidate their structure and function during the course of reaction, and to be able to build upon this knowledge. Operando investigations of amorphous materials have been invaluable in shedding light on such catalysts' operation. Key operando techniques include the use of vibrational (Raman and infrared) spectroscopy, UV-vis absorption spectroscopy, X-ray absorption spectroscopy, nuclear resonant inelastic X-ray scattering spectroscopy, X-ray diffraction analysis, X-ray photoelectron spectroscopy, in-line mass-spectrometry and gravimetric measurements. Each of these measurements provides complementary points of evidence (for example, the catalysts' oxidation state, composition, crystalline phase and surface intermediates) towards piecing together a complete picture of the material and reaction of interest.

**Fig. 1:**
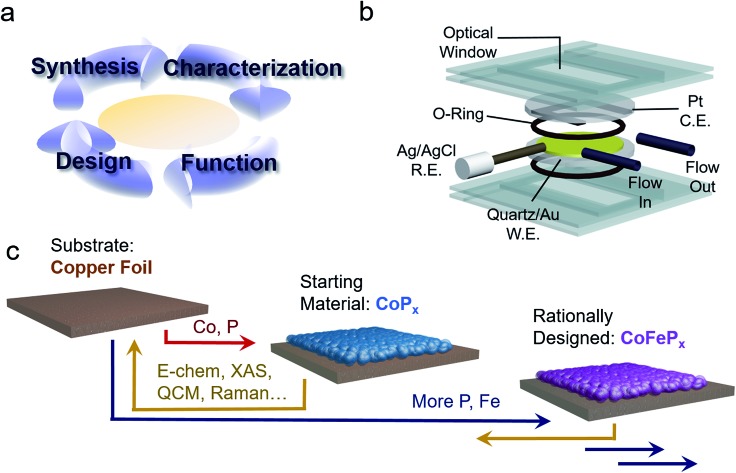
Strategy to develop a bifunctional water splitting catalyst with enhanced performance. An iterative cycle of synthesis, characterization, analysis, and design was employed to develop improved catalysts (a). Operando measurements of the catalyst were enabled by a QCM setup, which allows for integrated electrochemical, gravimetric, and spectroscopic analysis (b). A bifunctional HER and OER CoP_*x*_ catalyst was chosen as our starting material and improved upon using structure–function relationships derived through operando measurements to yield a rationally designed CoFeP_*x*_ (c).

To this end, we present the framework for a cycle of synthesis, characterization, function, and design utilizing operando Raman spectroscopy, electrochemical analysis, gravimetric measurements and a newly established quartz crystal microbalance (QCM) spectroelectrochemical cell (Fig. 1a and b). We apply this iterative cycle to amorphous cobalt phosphide (CoP_*x*_), which has previously been identified to catalyze both HER and OER in alkaline conditions with high activity.^10^–^13^ CoP_*x*_ is also simple to synthesize and the low cost of its components renders it a promising material for further development. Furthermore, bifunctionality allows for additional cost savings as well as an intrinsic stability over a much greater voltage range (in this case as the catalyst is stable throughout the OER and HER operational range) and the possibility to extend the lifetime of the catalysts through *in situ* electrochemical regeneration by bias-switching.^14^,^15^ With regards to scientific interest, bifunctionality hints towards reaction-dependent changes in surface composition and/or arrangement, further pressing the need for operando investigations. In this work, we carried out a combination of *ex situ* and operando measurements on CoP_*x*_ throughout its functional voltage range to capture its catalytically active forms. After identifying the active states of CoP_*x*_ as P-modulated metallic cobalt (HER) and a layered cobalt oxyhydroxide/dioxide (OER), we devised strategies to modify the material to enhance the activity of the catalytic sites. Doing so, we developed a second generation CoFeP_*x*_ catalyst through systematically adding P and Fe to enhance activity of the HER and OER active sites, respectively (Fig. 1c). Our 2^nd^ generation CoFeP_*x*_ surpasses the original CoP_*x*_ in both HER and OER activity and carries out overall water splitting at 1.5 V with 10 mA cm^–2^ in 1 M KOH electrolyte solution.

## Results and discussion

**Fig. 2:**
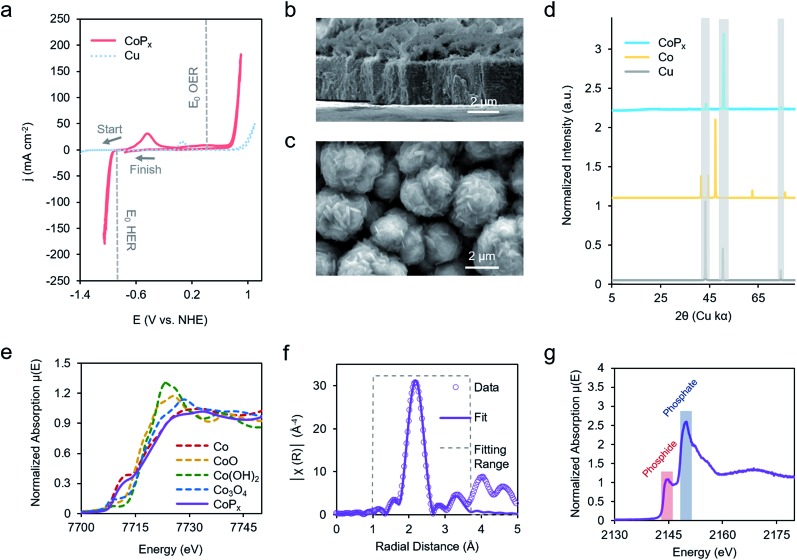
*Ex situ* characterization of CoP_*x*_ catalyst. CV scans of CoP_*x*_ electrodeposited on a Cu foil in 1 M KOH electrolyte solution. The standard potentials for HER and OER are also indicated by dashed lines assuming a pH of 14 (a). SEM images show that the CoP_*x*_ consists of a 4 mm thick film with a rough surface morphology (b and c). The XRD spectrum displays solely peaks attributed to the underlying copper substrate, pointing to the amorphous nature of CoP_*x*_ (d). XAS measurements, acquired pre-catalysis, illustrated that the Co component of CoP_*x*_ is similar to elemental Co (e). EXAFS fitting elucidated the local environment of Co, which closely resembled elemental Co, with the addition of P and O components (f). XAS measurements of P fraction signified that the phosphorus was present as a mixture of phosphide and phosphate (g).

Our chosen material of interest was amorphous CoP_*x*_,^10^,^11^,^13^,^16^ a known catalyst and promising starting point for further improvement. CoP_*x*_ was reductively electrodeposited from an aqueous solution of cobalt chloride, sodium hypophosphite, and sodium acetate through a series of cyclic voltammetry (CV) scans onto a copper foil substrate (Fig. S1†). Following the deposition, catalytic activity was recorded in an alkaline KOH electrolyte solution (1 M). CoP_*x*_ exhibited both HER and OER catalytic currents, requiring overpotentials of approximately 100 and 370 mV to reach 10 mA cm^–2^, respectively (Fig. 2a) (plotted using the projected surface area). 10 mA cm^–2^ is used as a benchmark current density because that is an estimated value at which a practical solar to fuel technology will have to operate and is a common reference point for comparison across catalysts in the literature.^17^ Note that under extremely acidic/basic conditions there is uncertainty on the exact pH value and therefore the thermodynamic potential of HER and OER. Thus, overpotentials for the half-reactions are only estimates assuming pH 14. However, performing CV to catalyze both reactions internally standardizes the measurements because the thermodynamic difference between the two half reactions will be fixed to 1.23 V, independent of the precise pH value. Prolonged chronopotentiometric scans revealed little activity decrease of the catalyst over the course of ∼17 hours, indicating excellent stability (Fig. S2†). Scanning electron microscopy (SEM) images (Fig. 2b and c), acquired after CV characterization, indicate the rough, flake-like structure of the CoP_*x*_ film with approximately 3–5 μm thickness. Energy dispersive X-ray spectroscopy (EDS) measurements, also acquired after CV testing of CoP_*x*_, point to an elemental composition of approximately 96.5% Co and 3.5% P. Low concentrations of P are common in electrodeposited cobalt–phosphorus containing films.^10^,^11^,^13^,^16^,^18^ The absence of any peaks beyond those belonging to the underlying copper foil in the powder X-ray diffraction (XRD) spectra of the CoP_*x*_ showed a lack of detectable long-range structure (Fig. 2d), consistent with the amorphous nature of this material.

In order to obtain a better understanding of this material we carried out a series of X-ray absorption spectroscopy (XAS) measurements on the CoP_*x*_ catalyst following its synthesis. Results from cobalt K-edge X-ray absorption near edge spectroscopy (XANES) of CoP_*x*_ were first compared to a series of Co containing standards (Fig. 2e). The spectral similarity and edge energy of CoP_*x*_ to Co signifies that the electronic structure and oxidation state component was close to Co(0), albeit with a less steeply rising edge (Fig. S3†).

Next, extended X-ray absorption fine structure (EXAFS) spectra of the Co were acquired and subsequently modelled. The fitting revealed that the 1^st^ coordination shell contained 9 Co nearest neighbors (in contrast to 12 for elemental Co) and ∼1–2 each of O and P atoms (Fig. 2F, S3 and Table S1†). A 2^nd^ shell of 3 Co atoms (bulk Co features 6) was also incorporated to attain a better fit. The EXAFS data suggests a local environment very close to elemental Co with a hexagonal close packed (HCP) crystal structure. Intensity in the EXAFS spectra at 4–5 Å suggests that some medium range order is retained within the CoP_*x*_ film through not at a length scale to yield peaks in the XRD spectrum. XANES spectra of P, on the other hand, indicated CoP_*x*_ featured a mixture of phosphide (peak at 2144 eV) and phosphate (peak at 2150 eV) incorporated within the amorphous framework (Fig. 2g).^19^,^20^ X-ray photoelectron spectroscopy (XPS) data (Fig. S4†) also points to the metallic nature of Co and mixture of phosphide and phosphate character of P in CoP_*x*_.^21^,^22^

Taken together, the data suggests that CoP_*x*_ consists of several shells of local ordering of HCP structured metallic cobalt atoms doped with P. XAS analysis of CoP_*x*_ revealed a high degree of similarity of the structural and electronic properties of CoP_*x*_ to Co, though the HER performance between the two materials differed greatly (*vide infra*). Second, the localized structural similarity of CoP_*x*_ to elemental Co is quite different than in other amorphous materials, such as electrodeposited CoO_*x*_ which featured a layered cobalt oxide structure,^23^ and CoS_*x*_,^24^,^25^ CoPi,^26^ Ni–B_*i*_,^27^ and MoS_*x*_,^28^ which exhibit typically 4–7 fold heteroatom coordination. In the case of CoP_*x*_, the small quantity of P likely functions as a dopant within a metallic-like Co matrix. This is also significantly different than crystalline cobalt phosphide catalysts (*i.e.*, CoP, CoP_2_, Co_2_P, *etc.*), which feature much higher proportions of P.^29^–^31^

Following initial *ex situ* characterization, we studied the operando structure and function of CoP_*x*_ with a combination of electrochemical, gravimetric, and spectroscopic measurements. To the best of our knowledge, this is the first time that this combination of measurements has been carried out to probe an electrocatalyst. Complementary multi-modal analysis is often necessary to piece together a complete picture of a material's precise structure, especially for the case of amorphous catalysts that are particularly prone to environment- and input-driven transformations and typically do not feature a well-understood *ex situ* structure as a starting point. In this work, QCM elucidated whether surface or bulk changes were occurring while Raman provided insights into the chemical nature of the metastable phases.

QCM offers exceptionally sensitive measurements of mass change on a piezoelectric quartz resonator substrate by recording mass-dependent changes in its resonance frequency under an alternating voltage.^32^,^33^ Briefly, the application of a voltage on a piezoelectric material such as quartz induces a physical deformation, which may be a cyclical deformation under an alternating current. The resonance frequency of the piezoelectric crystal, used as the working electrode in an electrochemical QCM experiment, is linearly dependent on the mass adsorbed onto its surface, and this quantity is used to aid in monitoring the behavior of catalysts deposited on the crystal. By comparing *in situ* changes in mass to the total mass deposited on the substrate, information from QCM measurements can help deconvolute surface from bulk transformations. However, it must be noted that chemical and electronic changes to the surface may also be interconnected with changes to the bulk.^34^

Raman spectroscopy, on the other hand, allows for the detection of certain molecular vibrational modes within both the bulk and surface of our material in the course of its function. Operando Raman measurements are also critical as post-electrolysis spectroscopy only captures an air-stable phase, which is not necessarily the catalytically active one.

**Fig. 3:**
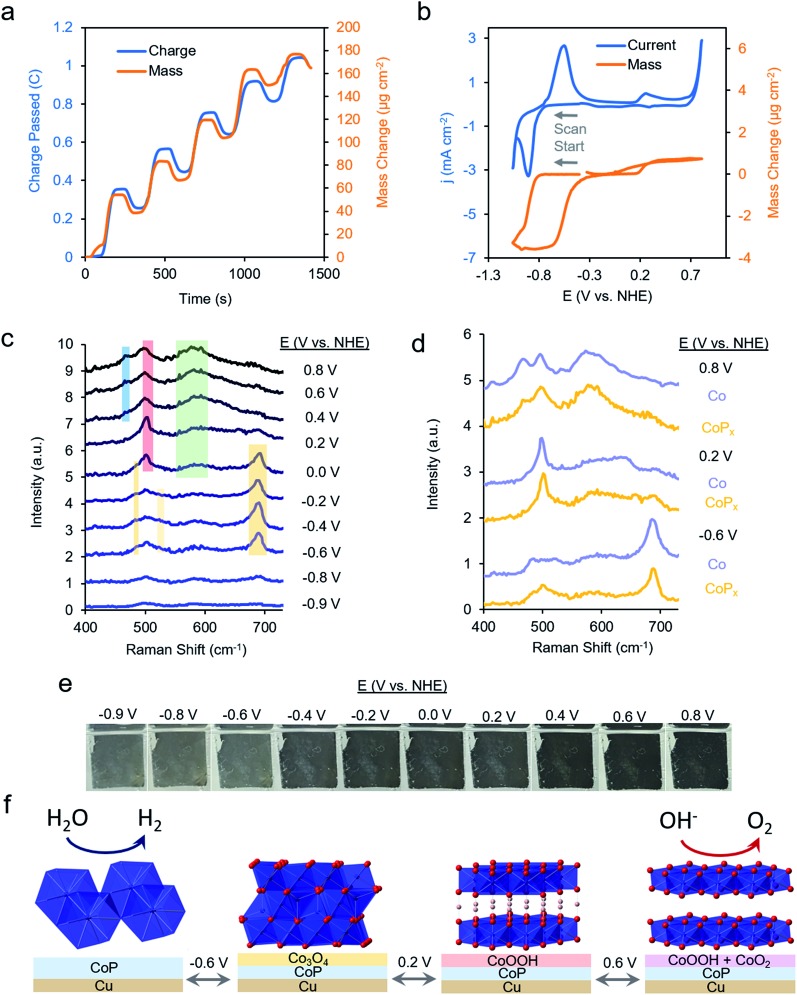
Operando characterization of CoP_*x*_. Quartz crystal microbalance – Raman spectroelectrochemical studies enable operando monitoring of mass change and molecular vibrations during an electrochemical measurement. The mass of CoP_*x*_ deposited on the working electrode was quantified during 5 CV cycles (a). A CV scan in 1.0 M KOH of CoP_*x*_ reveals a reversible change of ∼2.0% of the total mass corresponding to a redox wave centered at –0.8 V *vs.* NHE and a reversible mass change of 0.3% at 0.2 V *vs.* NHE (b). Operando Raman spectroscopy, also conducted in 1.0 M KOH at 25 °C, reveals the formation of Co_3_O_4_ from –0.6 V to 0.0 V *vs.* NHE, CoOOH at 0 V *vs.* RHE and CoO_2_/CoO_*x*_ at 0.4 V *vs.* NHE (c). The Raman spectra for CoP_*x*_ are similar to those of an electrodeposited Co under the same conditions, indicating that the spectra of CoP_*x*_ stem from phases of Co (d). An electrochromic effect (e) of the CoP_*x*_, measured on a fluorine-doped tin oxide electrode, also supports our assignment to the film interconversion between metallic, oxide, and oxyhydroxide phases. The dominant surface phase under each voltage range is illustrated in (f). Note: ions and solvent molecules filling in spaces between layers are not shown.

We first repeated the electrodeposition of CoP_*x*_ in our QCM cell under a constant flow of precursor solution. The buildup of CoP_*x*_ onto a gold-coated quartz chip by CV cycles under reductive potential was quantitatively monitored and yielded a value of 165.2 μg cm^–2^ after 5 cycles (Fig. 3a). This mass loading would correspond to an average film thickness of ∼0.2 μm by assuming a close-packed metallic Co composition (8.9 g cm^–3^), though the actual thickness is likely larger due to porosity and roughness effects.

The same sample's HER and OER catalytic activity was subsequently probed in 1 M KOH (Fig. 3b). Scanning towards cathodic potentials, a redox wave centered at –0.8 V *vs.* NHE corresponding to a reversible 2% (3.5 μg cm^–2^) mass loss/gain was observed prior to HER catalysis. Once HER catalysis began, the mass remained constant. Scanning the reverse direction, at anodic potentials, before the onset of OER catalysis, a broader redox wave at 0.3 V *vs.* NHE was observed. This wave corresponds to a reversible 0.3% (0.5 μg cm^–2^) mass gain/loss. Relatively small and fully reversible mass changes in comparison to the total catalyst amount point to a surface process occurring rather than a complete bulk transformation within CoP_*x*_. The measured mass changes were also reversible over multiple CV cycles. Furthermore, because the mass change plateaued prior to OER and HER catalysis, the formation of H_2_ and O_2_ gas would not significantly contribute to our observed QCM data in Fig. 3b.

Operando Raman spectra, acquired in the same cell, granted complementary information on the potential-dependent behavior of CoP_*x*_. In the reductive regime of –0.9 to –0.8 V *vs.* NHE, CoP_*x*_ yielded no distinct peaks in the Raman spectrum. At –0.6 V *vs.* NHE, spectral features attributed to Co_3_O_4_ arise, mainly in the form of a prominent band centered at 690 cm^–1^.^35^–^37^ Next, beginning at 0 V, the 690 cm^–1^ band decreases in intensity and a strong band at 503 cm^–1^ appears. This matches the dominant Raman active mode of CoOOH, indicating the conversion of surface Co_3_O_4_ to CoOOH.^36^,^38^ Further spectral changes were observed when applying potentials >0.2 V: the 503 cm^–1^ peak broadens and a wide feature around 580 cm^–1^ grows in intensity. A lone broad feature at 580 cm^–1^ was previously attributed to amorphous CoO_*x*_.^11^,^39^–^41^ At potentials of 0.6 and 0.8 V, the 580 cm^–1^ feature grows in intensity and another peak appears at 465 cm^–1^ as the 503 cm^–1^ peak decreases but does not fully fade. A coexistence of two broad peaks at 580 cm^–1^ and 465 cm^–1^ closely matched that of a weakly ordered CoO_2_ species.^42^–^45^ We propose here that under OER relevant voltages the surface is comprised of a mixture of CoOOH and CoO_2_ functioning as the OER active phase.

We compared the potential-dependent Raman spectra of CoP_*x*_ to those of an electrodeposited, P-free, cobalt film. The potential-dependent spectra of the cobalt film in the same conditions were nearly identical Raman spectra as CoP_*x*_, suggesting that both CoP_*x*_ and the similarly electrodeposited Co transverse through the same potential-dependent phases (Fig. 3d). The ∼35 cm^–1^ full-width half-max (FWHM) of the primary features in the Raman spectra indicate that the surface oxide domains have limited order and are nanoscopic (∼5 nm) in size.^46^ In addition to the spectroscopic and gravimetric data presented, we observed an electrochromic effect in CoP_*x*_ (Fig. 3e), in which the film displayed potential dependent coloration when moving from negative to positive potentials (between silver, grey, and black, negative to positive potentials), matching previous observations of electrodeposited Co films exhibiting electrochromic changes as they interconvert between metallic, oxide, and oxyhydroxide phases.^47^–^50^

Attenuated total reflection infrared spectroscopy (ATR-IR) analysis of post-electrolysis CoP_*x*_ imparted another layer of mechanistic information behind the material's potential-dependent behavior (Fig. S6†). The spectrum is largely featureless at –0.9 V *vs.* NHE, matching the expected behavior of a metallic cobalt film. At potentials positive of –0.4 V, peaks in the ∼1147 cm^–1^ range attributed to surface phosphates were observed, indicating that they stem from the oxidation of phosphide and are likely intermixed within the oxide surface layer.^51^ At –0.4 V, the lack of a band at 450 cm^–1^ and strong features at ∼500–650 cm points to Co_3_O_4_ being the dominant phase, as opposed to CoO.^52^ As the potential is further increased to 0.2 V and above, strong features in the 650–800 cm^–1^ range point to the rise of an oxyhydroxide phase.^53^

From the above data, a picture of the potential-dependent CoP_*x*_ behavior emerges (Fig. 3f). Before reaching HER catalysis, CoP_*x*_ reduces its oxide surface layer, which leads to a mass loss and the disappearance of the cobalt oxide Raman features. Reduction to metallic Co at –0.8 V *vs.* NHE can be expected from the cobalt-water Pourbaix diagram.^54^ The cobalt, as evidenced from XRD and XAS studies, has a close range HCP-like structure, with only short range order. The HER catalytic current greatly decreases upon the addition of 100 mM KSCN (Fig. S7†), an inhibitor of metal sites, suggesting cobalt to be in the metallic form and the primary HER active site here.^55^ In our case, we suggest that the role of the phosphorus component in CoP*_x_* may be to modify the electronic structure of the metallic cobalt active sites. This would modify the binding energy of hydrogen to them: a critical parameter influencing the HER catalytic activity.^56^

However, metallic cobalt is no longer at the surface as the potential is increased in the positive direction. Previous studies of crystalline transition metal phosphide OER catalysts have observed a thin oxygen-containing surface layer after water oxidation catalysis with *ex situ* transmission electron microscopy measurements.^57^,^58^ The CoP_*x*_ catalyst features similar electrochemical redox waves to an electrodeposited cobalt film (Fig. S8†) and matches the CV behavior that has been observed in electrodeposited cobalt films, with redox peaks assigned to interconversions between metal, oxide, hydroxide and oxyhydroxide phases.^47^–^50^ The OER-active species of CoP_*x*_, as deciphered through our multi-modal operando analysis, is ∼5 nm domains of layered CoOOH and CoO_2_ that grow together overtop the conductive CoP_*x*_ bulk material. The OER active sites in basic conditions have previously been suggested to be surface-exposed Co(iii) and Co(iv) sites of CoOOH and CoO_2_.^23^,^50^,^59^–^64^ These materials feature edge-sharing CoO_6_ octahedra, oriented in a layered structure with accessible interlayer sites for OER catalysis. This accessible surface, along with the small domain size, leads to exposure of a high density of crystalline faces, edges and corners to the electrolyte. The exposed surface is thought to be key behind the high OER performance of this material.^62^ In brief, operando QCM studies revealed that a reversible surface process was occurring and operando Raman, in conjunction with *ex situ* measurements helped elucidate the chemical nature of the surface-active species, which would have been difficult to capture with a single bulk characterization technique (*e.g. in situ* EXAFS).

Inspired by the ability to capture the dynamic nature of CoP*_x_* at the molecular scale, we moved to build upon these insights to design an improved next-generation catalyst. Our experiment elucidated the active state of CoP_*x*_ under HER and OER operating conditions; we thus devised rational strategies of modifying the catalyst to improve the activity of the active species. Because the HER-active state was deemed likely to be P-doped metallic Co, we reasoned that systematically modifying the P content within the material would lead to an altered electronic structure of the Co active site. This would consequently lead to modified binding energies of the adsorbed hydrogen intermediates and thus, enhanced or diminished HER catalytic activity.^56^

**Fig. 4:**
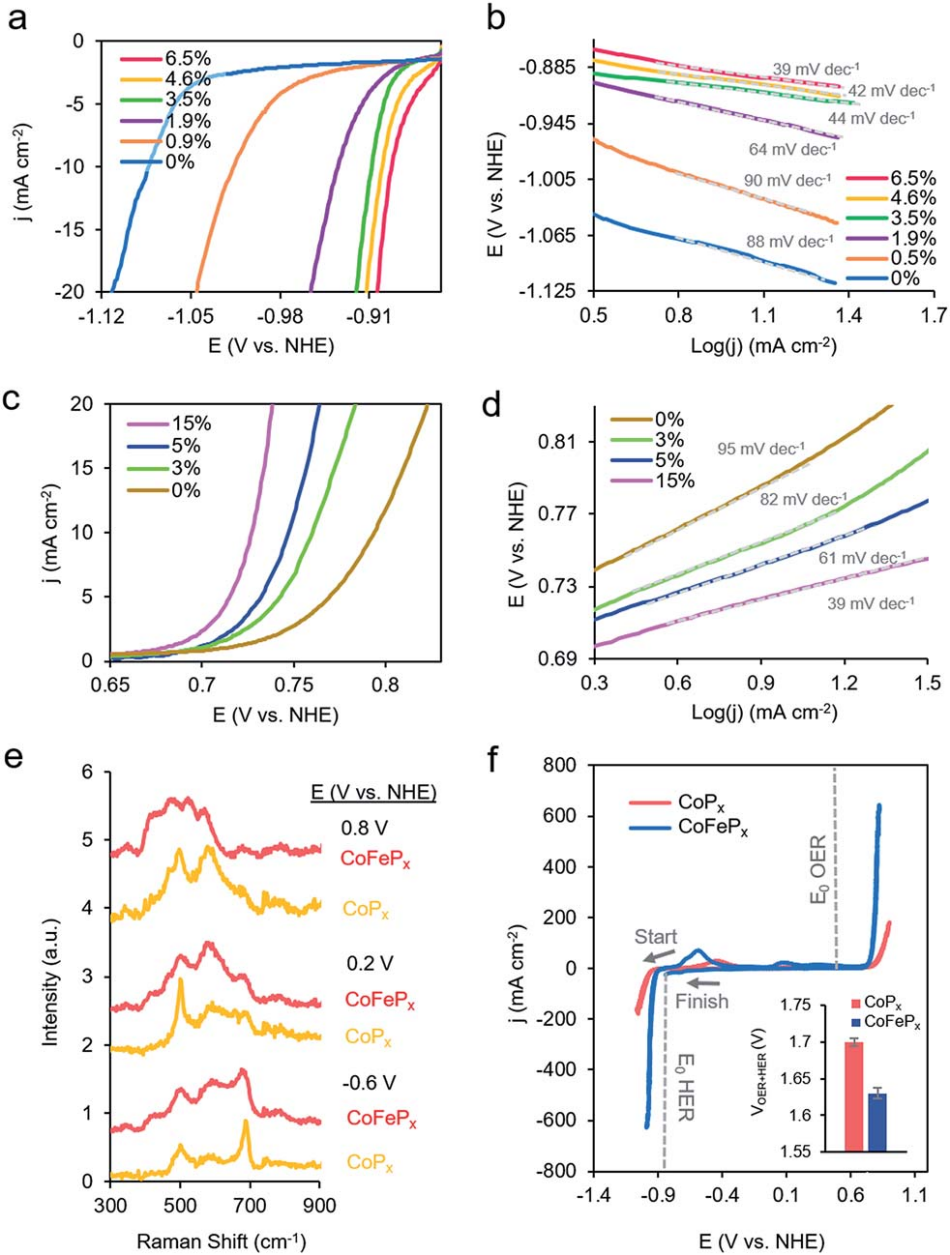
Systematically enhanced performance of bifunctional catalyst guided by insights from operando measurements. CV illustrated that increasing P doping improves the HER performance (a) and decreases the Tafel slopes (b). Iron doping was utilized to improve the OER kinetics (c) with a concomitant decrease in Tafel slopes (d). The optimized CoFeP_*x*_ catalyst displayed spectroscopically distinct Raman active vibrations, which do not perfectly match any cobalt or iron phases but rather resemble cobalt–iron mixed-metal oxide phases (e). The CoFeP_*x*_ catalyst outperforms the first generation CoP_*x*_ in both OER and HER activity (f). The inset depicts the total voltage necessary to achieve 10 mA cm^–2^ for overall water electrolysis for CoP_*x*_ and CoFeP_*x*_. All measurements were conducted in 1.0 M KOH at 25 °C.

To test this hypothesis, we synthesized a series of CoP_*x*_ catalysts with varying amounts (0 to 3 M) of sodium hypophosphite in the solution and subsequently varying amounts of P incorporated (0 to 6.5 atomic%; further increasing hypophosphite concentration did not result in increased P incorporation), then recorded their catalytic activity (Fig. 4a). We discovered that the HER activity increased and the Tafel slopes decreased with systematically increasing P that was incorporated into the film (Fig. 4a, b and S9†). As metallic cobalt HER activity is limited by a high Co–H binding energy that is too high,^56^ increasingly incorporating electronegative P dopants into the lattice likely weakens this bond, shifting it closer to being endergonically neutral and consequently increasing HER kinetics.^65^,^66^ Furthermore, in alkaline conditions, as water is the primary proton source, the first reaction step in the HER consists of water dissociation. Electronegative dopants such as P would also give rise to more positively charged Co active sites, which may exhibit increased electrostatic affinity to OH^–^ species generated by water dissociation, thereby decreasing the barrier for the water dissociation step, and consequently, further accelerating HER kinetics.^67^–^69^

The presence of only surface oxides/hydroxides is believed to be a minor contributor, by themselves, to the enhancement of the HER reaction because of the relatively poor performance of an equivalent P-free electrodeposited cobalt film towards the HER. Given the fact that the P content is still relatively minor, we believe that the amorphous nature and active sites on the surface are similar as for the first generation CoP_*x*_, with the catalytic enhancement stemming from slight electronic differences of the cobalt species. Indeed, when comparing the high-resolution Co 2p XPS data of CoP_*x*_ samples with 0.9% P and 6.5% P, a decreased magnitude of satellite peaks in the spectra of the latter point to a more cationic state (less valence electrons) of Co in CoP_*x*_ 6.5% P (Fig. S10†).^21^ The OER activity exhibited little change in this process (Fig. S9†), suggesting that increasing P content did not significantly change the nature of the oxide/oxyhydroxide layer formed under anodic conditions. The cumulative effect of P incorporation decreased the total voltage necessary to attain 10 mA cm^–2^ for water electrolysis with the bifunctional catalyst from 1.84 V to 1.67 V for 0 and 6.5% P containing films, respectively (Fig. S9†).

We subsequently shifted our focus towards improving the OER activity of our CoP_*x*_ catalyst. As we had established that the OER active phase was the layered CoOOH and CoO_2_, our strategy of choice to improve the OER active material was through Fe doping to generate the mixed-metal oxide/oxyhydroxide which retained the layered structure and active site accessibility, yet benefited from favorable effects of Fe doping. Incorporation of Fe sites into Co- and Ni-based oxide OER catalysts has been previously demonstrated to enhance OER catalysis due to the promotion of higher valent Co sites due to Fe Lewis acidity, by incorporating highly active Fe sites in a conductive Co or Ni matrix, or by modifying interfacial interactions between solvent, intermediates, and active sites, though an agreed-upon mechanism is lacking.^70^–^76^

Beginning with the best performing HER material (6.5% P), we incorporated Fe into CoP_*x*_ by immersing the catalyst into an 80 °C aqueous solution of FeCl_2_ (0.1 M) for selected amounts of time (30 s to 60 min). The resulting films (Fig. S11†) were denoted by their overall Fe *vs.* Co atomic composition, though the Fe was more concentrated near the surface (Fig. S12†). Immersion times beyond 60 min did not increase the Fe concentration. Systematically increased Fe incorporation resulted in increased OER activity and decreased Tafel slopes (Fig. 4c and d). The incorporation of Fe into the material led to only small decreases in HER activity, which were outweighed by the gains made on the OER end (Fig. S13a†). The incorporation of Fe led a decrease in water oxidation overpotential at 10 mA cm^–2^ by ∼60 mV and an overall water electrolysis voltage decrease from 1.67 V to 1.63 V at 10 mA cm^–2^ (Fig. S13b†).

While the operando mass change for CoFeP_*x*_ was similar to CoP_*x*_ (Fig. S14†), Raman spectroscopy revealed a distinct set of bands under reaction conditions (Fig. 4a and S15†). At –0.6 V *vs.* NHE, the spectrum resembled that of Co_3–*x*_Fe_*x*_O_4_, primarily evidenced through a widened and blue-shifted band at ∼677 cm^–1^.^77^–^79^ This material also features a spinel-like crystal structure similar to Co_3_O_4_ observed for CoP_*x*_ under similar voltages. The CoFeP_*x*_ spectrum continued to evolve at more positive voltages, with the ∼677 cm^–1^ band being diminished and peaks arising in the 420–580 cm^–1^ range. Co_3–*x*_Fe_*x*_O_4_ polarized at oxidizing potentials and CoFe layered double hydroxides have Raman features centered in this range.^80^,^81^ Furthermore, we do not observe Raman features associated any pure iron oxide/oxyhydroxide phase, indicating a lack of obvious phase segregation.^82^,^83^

Post-electrolysis IR spectra (Fig. S16†) point to interconversions between a cobalt–iron mixed-metal spinel (bands at 499 cm^–1^ and 623 cm^–1^), to a layered (oxy)hydroxide (band at 846 cm^–1^).^84^–^86^ Shifted band positions and altered potential-dependent spectral transitions relative to what was observed for CoP_*x*_ provide further evidence of Fe substitution within the surface domains. Given the whole of the data, we believe that the mixed-metal (oxy)hydroxide OER active phase of CoFeP_*x*_ also retains the layered structure with a high amount of accessible surface area and additional electronic and catalytic benefits conferred by the added-in iron species. The end-result of our iterative cycle is presented in Fig. 4f. For a benchmark 10 mA cm^–2^ for overall water electrolysis, a net gain of ∼210 mV over electrodeposited Co and ∼70 mV over CoP_*x*_ was attained (Fig. 4f inset). The CoFeP_*x*_ displayed stability for >200 h under chronopotentiometric measurements (Fig. S17†). Surface regeneration through bias switching may be applied for even longer operating lifetimes in commercial applications.^14^,^15^

The faradaic yields for H_2_ and O_2_ for CoFeP_*x*_ were quantitative (Fig. S18a and b†). In addition, the yield for H_2_ under an operating current of 10 mA cm^–2^ was not significantly diminished in the presence of oxygen, when running electrolysis in a 1-compartment cell and decreased to ∼90% in a fully air-saturated electrolyte (Fig. S19†). We refer the interested reader to a review on oxygen tolerant catalysis for further insights.^87^ Overall, this represents, to the best of our knowledge, the first example of systematically improved activity for both reactions of a bifunctional catalyst using insights gained through operando characterization.

**Fig. 5:**
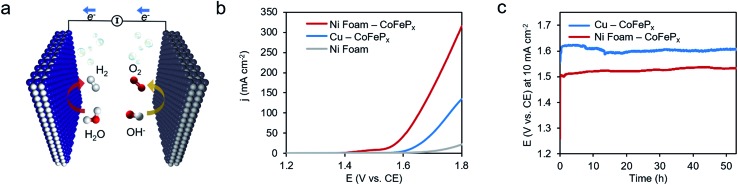
High performance water electrolysis. Overall water electrolysis with the optimized CoFeP_*x*_ catalyst as the cathode and anode (a). Linear sweep measurements show the onset of water electrolysis at ∼1.6 V for CoFeP_*x*_ deposited on planar copper and ∼1.5 V when CoFeP_*x*_ is deposited on a high surface area nickel foam substrate (b). The nickel foam contribution is negligible in this range. A voltage of 1.62 V and 1.50 V was necessary to drive the overall water electrolysis at 10 mA cm^–2^ for CoFeP_*x*_ on copper and nickel foam, respectively, and did not significantly increase over the course of 50 hours (c). All measurements conducted in 1.0 M KOH at 25 °C.

Finally, we demonstrate the excellent performance of the CoFeP_*x*_ catalyst in alkaline electrolysis for overall water electrolysis measurements in a 2-electrode configuration (Fig. 5a). CoFeP_*x*_ on a planar copper foil required ∼1.6 V to attain 10 mA cm^–2^ towards overall water splitting (Fig. 5b). In order to compare to commercial electrolyzers, which commonly take advantage of high surface area substrates, CoFeP_*x*_ was also deposited on a nickel foam substrate (Fig. S21–S24†). Due to an increased quantity of active sites per projected area, both the current densities of HER and OER were increased (Fig. S21–S24†).

In a 2-electrode configuration, the net effect was a decrease in total voltage to ∼1.5 V to reach 10 mA cm^–2^ (Fig. 5b). The bare nickel foam, a common material in alkaline electrolyzers, needed almost 1.8 V to match the equivalent current density. After 50 h of continuous operation, only minimal losses (∼30 mV) were experienced (Fig. 5c) in a demonstration for CoFeP_*x*_ practical applicability. The exact thermodynamic potential of HER and OER half-reactions is difficult to determine in extreme pH conditions, but our two-electrode experiments with the bifunctional catalyst give a unique opportunity to quantitatively gauge performance because the potential difference between the reductive and oxidative reactions is always constant. Therefore, the total overpotential (*η*_HER_ + *η*_OER_), with the inclusion of other resistance losses, can be exactly determined. Further approaching the operating conditions of typical alkaline fuel cells (10 M KOH at 80 °C), the CoFeP_*x*_ catalyst, deposited on Ni foam can drive an overall water electrolysis current of 100 mA cm^–2^ at 1.5 V (Fig. S25†). For reference, a comparison to the best performing bifunctional catalysts in the literature is reported in Table S2.†

## Conclusions

In conclusion, we present a study for catalyst development through a cycle of synthesis, characterization, analysis, and re-design. We investigated an amorphous cobalt phosphide bifunctional catalyst with an array of *ex situ* and operando methods to elucidate its behavior under operating conditions. Capturing the potential-dependent surface-active species with our multi-modal approach aided us in devising a strategy to rationally enhance the activity of those sites. Doing so, we designed a CoFeP_*x*_ material improving upon the original for HER and OER reactivity and capable of overall water electrolysis at 1.5 V in 1 M KOH electrolyte. Overall, the work presented here advances the understanding of amorphous materials and their catalytically active dynamics and interfaces. Furthermore, this set of techniques and analytical approach is also not restricted to water splitting catalysts and can benefit a wide range of catalytic reactions such as CO_2_ and N_2_ reduction.

## Experimental section

### Electrochemistry

The precursor solution for CoP_*x*_ consisted of sodium acetate (0.1 M), cobalt chloride (0.05 M), and sodium hypophosphite (1.0 M). The P content was modified by varying the sodium hypophosphite concentration (0, 0.03, 0.1, 0.5, 1.0, 3.0 M), while keeping the concentrations of the other precursors constant. CoP_*x*_ was electrodeposited through 16 CV cycles between –0.1 and –0.8 V *vs.* NHE onto a cleaned copper, nickel-foam, or fluorine-doped tin oxide (FTO) substrate, with a typical electrode area of 0.1 cm^–1^. Iron was doped into the CoP_*x*_ film by immersing the CoP_*x*_ post-synthesis into a solution of iron(ii) chloride (100 mM) at 80 °C for times of 30 s, 5 min, 15 min, and 60 min. Following synthesis, the substrates were thoroughly rinsed with water. A two-compartment cell was utilized, unless specified otherwise. For two-electrode overall water electrolysis measurements, experiments were conducted in a 1-compartment cell in a nitrogen atmosphere to minimize resistance between the two electrodes. In a three electrode configuration, a Ag/AgCl reference electrode and a glassy carbon rod counter electrode were utilized. All measurements were conducted at ∼25 °C. The catalytic activity of CoP_*x*_ and CoFeP_*x*_ films were studied a KOH electrolyte solution (typically 1.0 M) under a nitrogen atmosphere. Each film was tested for HER and OER activity in a CV scan (5 mV s^–1^), allowing for the total voltage of overall water electrolysis to be accurately recorded. IR compensation (∼1 ohm, determined through electrochemical impedance spectroscopy) was performed to account for solution resistance. Prior to each CV, the catalyst film was held at –0.8 V *vs.* NHE for 150 s to activate the HER active state. Chronopotentiometric stability scans were performed at +10 and –10 mA cm^–2^. Current densities were plotted using the projected surface area. The actual surface area is much greater and estimates of the actual surface area, leading to an enhanced activity are provided in Fig. S23 and S24.† Alvatek Ivium CompactStat and IviumStat potentiostats were used for electrochemistry experiments. Reference electrodes were tested after prolonged measurements to exclude effects of potential drifts on measurements.

### X-ray absorption spectroscopy

XAS data of CoP_*x*_ films were acquired at B18 beamline at Diamond Light Source in transmission and total electron yield mode. Energy calibration was performed using a series of standards (*e.g.* Co foil). CoP_*x*_ was electrodeposited onto a thin ∼250 μm class cover slip coated with FTO for optimal signal quality. Phosphorus XAS data were acquired in an oxygen-free chamber. XANES normalization and processing was performed with Athena software to remove pre- and post-edge contributions. Artemis, IFEFFIT and Feff 6 software were utilized to EXAFS data processing and analysis.^88^–^90^ EXAFS data were fit according the EXAFS equation:
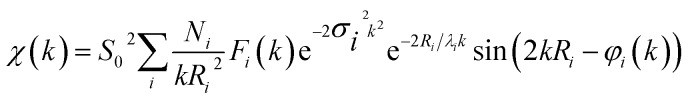


Experimental data were fit with *ab initio* phases and amplitudes used in the EXAFS equation to fit the experimental data. A Hanning window was utilized for the Fourier-transform of the data. In the fitting, *N*_*i*_ were coordination numbers of the central atom located at a distance of *R*_*i*_. *F*_*i*_(*k*) is the amplitude function of each shell. The amplitude reduction factor, *S*_0_^2^, is intrinsic to the central atom and accounts for shakeup processes. This value was 0.76 for Co and was held consistent throughout all fitting. The Debye–Waller term, e^–2*σ*_*i*_^2^*k*^2^^, factors in thermal and/or structural disorder. The mean free path term, e^–2*σ*_*i*_^2^*k*^2^^, where *λ*_*i*_, is the photoelectron mean free path, accounts for amplitude losses in inelastic scattering. The sinusoidal component: sin(2*kR*_*i*_ + *φ*_*i*_(*k*)), accounts for the periodicity in the X-ray absorption coefficient. Here, *φ*_*i*_(*k*) is the phase function for the coordination shell, *i*. Throughout the EXAFS fitting process, all variables were fixed except for *R*_*i*_, *N*, and *σ*_*i*_, which were allowed to float. Established crystal structures of hexagonal close packed cobalt metal, CoO, CoP, and Co_2_P were used as standard models for fitting. Additional EXAFS fitting is presented in Table S2, Fig. S27 and S28.†

### X-ray photoelectron spectroscopy

XPS spectra were taken using an ESCALAB 250Xi instrument. Prior to data acquisition, the surface of each sample was etched away to remove contaminants and any native oxide layers. Quantitative peak fitting was performed after a Shirley-type function removed the background arising from energy loss. Following this, processed spectra were fitted to a series of Voight-type peaks to take in consideration spectrometer (Gaussian) and lifetime (Lorentzian) broadening.

### Electron microscopy

Scanning electron microscopy was performed with a FEI Philips XL30 FEG SEM. Imaging was performed at 5 keV while EDS analysis was performed at 20 keV, which captured mainly bulk composition due to the electron beam penetration depth at this voltage. EDS line scan cross-section analysis was acquired at a 90 degree tilt.

### Quartz crystal microbalance measurements

QCM measurements were performed with a Biolin Q-sense explorer module. A custom QCM cell was used that allowed QCM measurements to be conducted simultaneously with optical measurements through a quartz top window. A gold coated quartz chip served as both a working electrode and piezoelectric sensor. All measurements were conducted in flow-mode. All harmonics gave similar signals during the measurements, and the 3^rd^ harmonic was used for quantification. Changes in mass were quantified using the Sauerbrey equation:
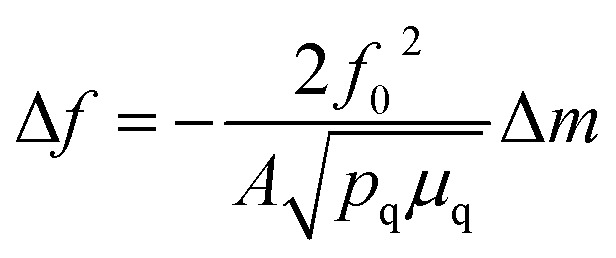
where *f*_*0*_ is the resonance frequency of the quartz oscillator, *A* is the piezoelectrically active crystal area, Δ*m* is the change in mass, *p*_q_ is the density of quartz, and *μ*_q_ is the shear modulus of quartz. Mass losses were reversible over multiple CV cycles, run at 5 mV s^–1^.

### Raman spectroscopy

A Horiba Jobin Yvon Labram HR Evolution confocal Raman instrument and Labspec software were utilized to acquire Raman spectra. Raman spectra were acquired with a 473 nm diode laser operating at 1 mW laser power. Sample integrity was confirmed by (1) comparing spectra at varying laser intensity and (2) by monitoring for any changes in spectral shape over time. Furthermore, during spectral acquisition, the sample was continually raster scanned to minimize exposure to any single area. To acquire spectra operando, the same cell was used as for QCM measurements. All Raman measurements were performed under steady state conditions in a stepped-chronoamerometry mode following QCM analysis. All spectral changes were reversible upon switching back to the original voltage. Experimental spectra of CoP*_x_* and CoFeP*_x_* were compared to those obtained from commercially available standards (Fig. S26†).

### Infrared spectroscopy

Attenuated total reflection Infrared (ATR-IR) spectra of CoP_*x*_ and CoFeP_*x*_ post-electrolysis samples were obtained by using a Thermo Scientific Nicolet iS 50 FT-IR spectrometer, equipped with a diamond single-reflection crystal, a DLaTGS detector and a Polaris™ IR radiation source. Spectra were acquired in the spectral region between 4000 and 400 cm^–1^ with a spectral resolution of 4 cm^–1^. Each spectrum is accumulated by 200 scans in total. ATR-IR spectra were evaluated using Bio-Rad Win-IR Pro software.

### Determination of faradaic yields

Headspace H_2_ was quantified using gas chromatography (Shimadzu GC equipped with a Mol-sieve column). GC calibration was performed with known quantities of H_2_ and CH_4_ to precisely quantify the response factors for each gas over the range of our experimental data and quantify their response factor ratio (H_2_/CH_4_ response factor) as well. CH_4_ was utilized as an internal standard to account for any potential leakage of gas out of the electrolysis cell.

Prior to electrolysis, the two-compartment reaction cell was purged with 2% CH_4_ in N_2_ as the internal standard for 30 minutes. At periodic intervals, gas extracted from the reactor and injected into the GC using a gas-tight syringe. Faradaic O_2_ yields were recorded using an Ocean Optics NeoFox Fospor fluorescent sensor, connected into the reaction cell through a fiber-optic cable, and Neofox Viewer software. Dissolved O_2_ was accounted for through Henry's Law.

## Conflicts of interest

There are no conflicts to declare.

## Supplementary Material

Supplementary information
