# Supplementary material for: Catalysis by design: development of a bifunctional water splitting catalyst through an operando measurement directed optimization cycle
Source: Chem Sci. 2018 May 8;9(24):5322–33. doi: 10.1039/c8sc01415a (PMC6009440; doi:10.1039/c8sc01415a)
Supplement: Supplementary file 1 [file SC-009-C8SC01415A-s001.pdf]

Electronic Supplementary Material (ESI) for Chemical Science.
This journal is © The Royal Society of Chemistry 2018

Electronic Supplementary Information

Catalysis by Design: Development of a Bifunctional Water Splitting Catalyst through an *In Operando* Measurement Directed Optimization Cycle

Nikolay Kornienko[a], Nina Heidary[a], Giannantonio Cibin[b] & Erwin Reisner[a*]

[a] Department of Chemistry, University of Cambridge, Lensfield Road, Cambridge CB2 1EW, U.K.

[b] Diamond Light Source Ltd., Diamond House, Harwell Science and Innovation Campus, Didcot OX11 0DE, U.K.

[*] To whom correspondence may be addressed. Email: reisner@ch.cam.ac.uk

<u>Table of Contents:</u>

Table S1. EXAFS fitting results 3
Table S2. Comparison of catalysts 4
Fig. S1. Electrodeposition of CoP_x catalyst 5
Fig. S2. Stability of CoP_x electrodes 6
Fig. S3. XANES and EXAFS comparisons of Co and CoP_x 6
Fig. S4. CoP_x XPS spectra 7
Fig. S5. Current and voltage traces from CoP_x deposition in QCM cell 7
Fig. S6. Post-electrolysis infrared spectra of CoP_x 8
Fig. S7. Inhibition of CoP_x HER current with KSCN 8
Fig. S8. Comparison of Co and CoP_x CVs 9
Fig. S9. Effects of P incorporation 9
Fig. S10. XPS analysis of CoP_x with different amounts of P 10
Fig. S11. SEM imaging of $CoFeP_x$ catalysts 10
Fig. S12. Elemental analysis of $CoFeP_x$ 11
Fig. S13. Effects of Fe doping 11
Fig. S14. QCM probing of $CoFeP_x$ catalyst 12
Fig. S15. *Operando* Raman probing of $CoFeP_x$ 12
Fig. S16. Post-electrolysis infrared probing of $CoFeP_x$ 13
Fig. S17. $CoFeP_x$ stability testing 13
Fig. S18. Product quantification 14
Fig. S19. H_2 quantification with and without O_2 present 14
Fig. S20. Comparison to Cu substrate 15
Fig. S21. Comparison to Ni foam substrate 15
Fig. S22. SEM imaging of $CoFeP_x$/Ni foam 16
Fig. S23. Characterization of $CoFeP_x$ on Ni foam 16
Fig. S24. Surface area estimation with capacitance measurements 17
Fig. S25. Overall water electrolysis under harsh conditions 17
Fig. S26. Raman spectra of commercially available standards 18
Fig. S27. EXAFS fitting data 18
Fig. S28. XANES characterization of $CoFeP_x$ 19
References 20

Element	Shell	N	R_{eff} (Å)	σ
Co	1	9.3 (0.5)	2.50 (0.05)	0.0057 (0.00032)
Co	2	3.0 (0.6)	3.54 (0.07)	0.0067 (0.00181)
P	1	1.8 (0.4)	2.20 (0.12)	0.0045 (0.00121)
O	1	1.5 (0.3)	2.13 (0.13)	0.0047 (0.00128)

k_{min}	k_{max}	R_{min}	R_{max}
2	12.5	1	3.7

R-Factor			
.00794			

Table S1: Values obtained from and parameters used for EXAFS fitting. The best fit was obtained using a first shell of predominantly cobalt nearest neighbors, with minor contributions of phosphorus and oxygen atoms. 1st shell indicates nearest neighbors directly bonded to the central atom. N indicates the coordination number, R_{eff} indicates the calculated bond length, and σ is the Debye-Waller factor. The subscript in grey is the error margin in the fitting. If keeping all other fitting parameters and variables consistent but removing P and O scattering paths, the R factor (goodness of fit) increases from 0.0079 to 0.0127.

Catalyst	Electrolyte	j (mA cm^{-2}	Voltage (V)	Notes	Reference
CoP_x – Cu foil	1M KOH	10	1.70	From chronoamperometry	This work
$CoFeP_x$ – Cu Foil	1M KOH	10	1.62	From chronoamperometry	This work
$CoFeP_x$ – Ni Foil	1M KOH	10	1.50	From chronoamperometry	This work
$CoFeP_x$ – Ni Foil	10M KOH	100	1.50	From chronoamperometry	This work
Co-P	1M KOH	4	1.63	From chronoamperometry	1
NiFeOx	1M KOH	10	1.51	From chronoamperometry	2
Ni-Co/1-T MoS_2	1M KOH	10	1.44	From chronoamperometry	3
Ni_5P_4	1M KOH	10	1.7	From linear sweep	4
Ni_2P	1M KOH	10	1.63	From chronoamperometry	5
MoO_2	1M KOH	10	1.53	From chronoamperometry	6
MoS_2/Ni_3S_2	1M KOH	10	1.56	From chronoamperometry	7
NiFe LDH	1M NaOH	10	1.7	From chronoamperometry	8
NiSe	1M KOH	20	1.74	From chronoamperometry	9
CoSe/NiFe LDH	1M KOH	20	1.71	From chronoamperometry	10
NiMo	1M KOH	10	1.64	From chronoamperometry	11
$NiCo_2O_4$	1M KOH	10	1.65	From chronoamperometry	12
Ni/Ni_8P_3	1M KOH	10	1.61	From chronoamperometry	13
CoP/RGO	1M KOH	10	1.7	From chronoamperometry	14
NiS	1M KOH	10	1.64	From chronoamperometry	15
Ni_2P	1M KOH	10	~1.55	From chronoamperometry	16
MoO_x/Ni_3S_2/NF	1M KOH	10	1.5	From chronoamperometry	17
CP@Ni-P	1M KOH	10	1.5	From chronoamperometry	18
$Co_3O_4/CoMoO_4$	1M KOH	10	1.5	From linear sweep	19
Ni-P	1M KOH	10	~1.7	From chronoamperometry	20
Cu@NiFe LDH	1M KOH	10	1.54	From chronoamperometry	21
VOOH	1M KOH	10	1.62	From linear sweep	22
Ni_3FeN	1M KOH	10	1.495	From linear sweep	23
FeMnP/graphene	1M KOH	10	1.6	From chronoamperometry	24
$SrNb_{0.1}Co_{0.7}Fe_{0.2}O_{3-\delta}$	1M KOH	10	1.65	From chronoamperometry	25
NC/CuCo/$CuCoO_x$	1M KOH	10	1.53	From chronoamperometry	26
Ni_xP_y	1M KOH	10	1.57	From chronoamperometry	27

Table S2: Comparison of bifunctional catalysts for overall water electrolysis in alkaline electrolyte solution. Note, that the projected surface area is used in this work and estimates of the actual surface area are provided in Fig. S24.

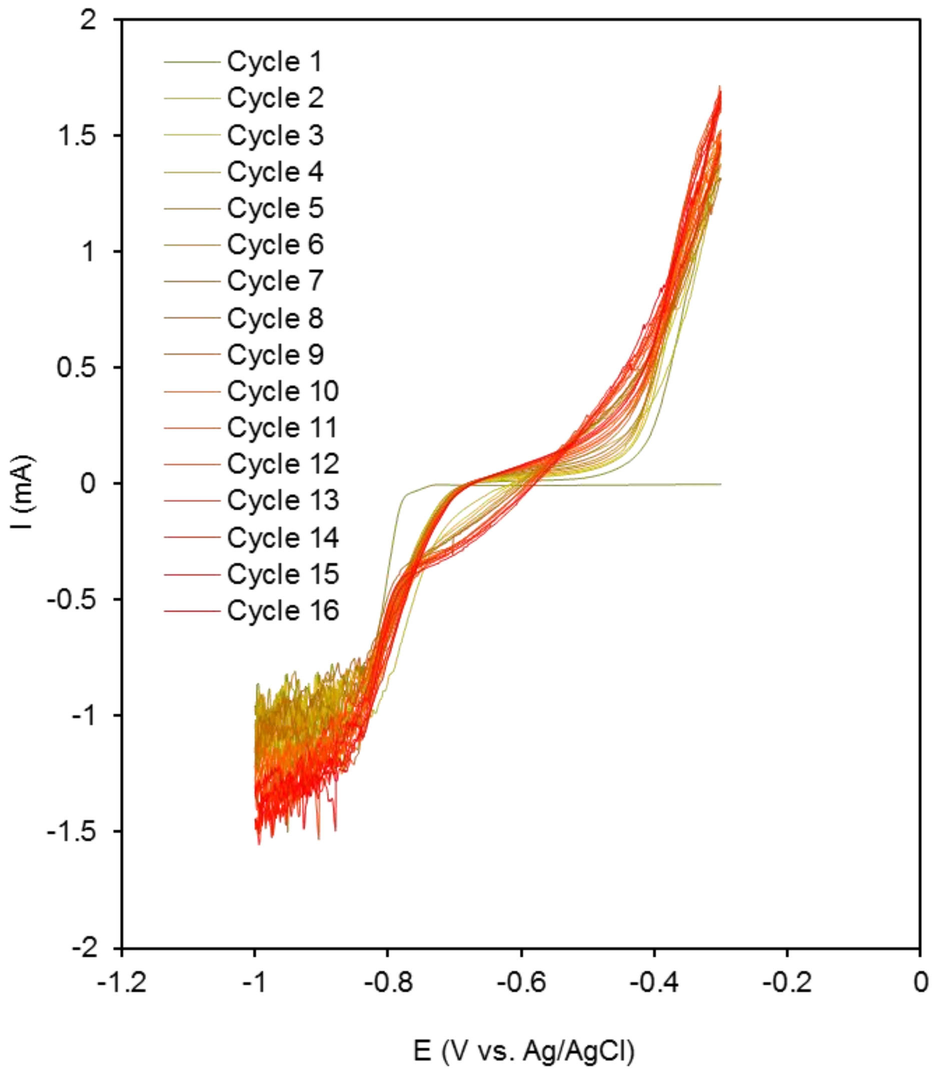

Fig. S1. Electrodeposition of the CoP_x catalyst. CoP_x was electrodeposited onto a copper foil through a series of CV cycles between –0.3 and –1.0 V vs. Ag/AgCl from a deposition solution consisting of $CoCl_2$ (50 mM), sodium acetate (0.1 M), and sodium hypophosphite (0.5 M). The deposition was conducted at 25° C and a glassy carbon rod was utilized as a counter electrode.

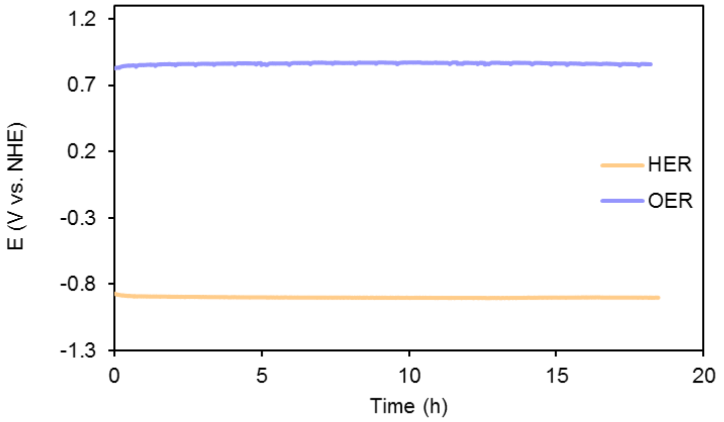

Fig. S2: Stability of CoP_x electrodes. CoP_x stability under HER and OER conditions was evaluated by chronopotentiometry at –10 mA cm^{-2} and +10 mA cm^{-2} in a KOH electrolyte solution (1 M). A three-electrode configuration with a Ag/AgCl reference and a glassy carbon electrode counter was employed. Nitrogen was continually purged throughout the solution during the course of measurement. The potential of the reference electrode was also checked before and after the measurement to make sure there was not a significant potential drift that could influence the chronopotentiometric data.

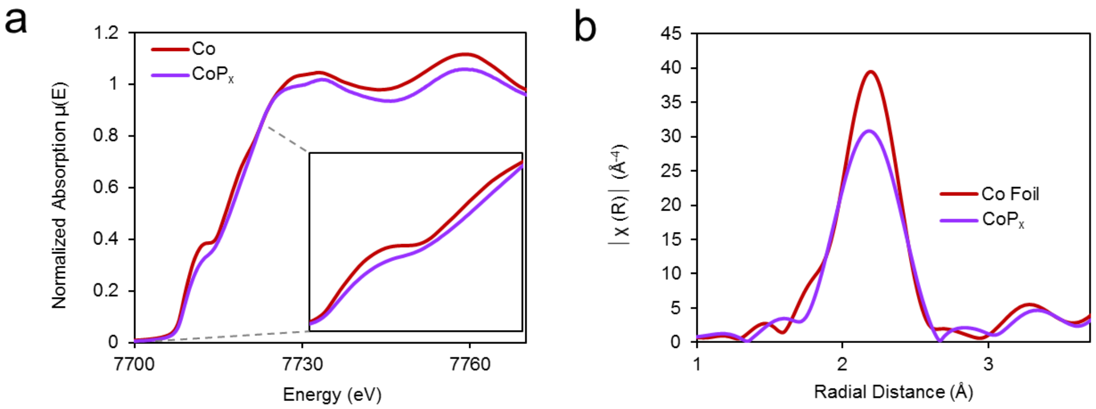

Fig. S3: XANES (a) and EXAFS (b) data of CoP_x and bulk Co. Slight differences in electronic structure are evident in a less steeply rising edge for CoP_x in the XANES data, while changes in local coordination in are evidenced through decreased amplitude of the first and second shell peaks in the EXAFS spectrum. However, the Co in CoP_x is still metallic-like.

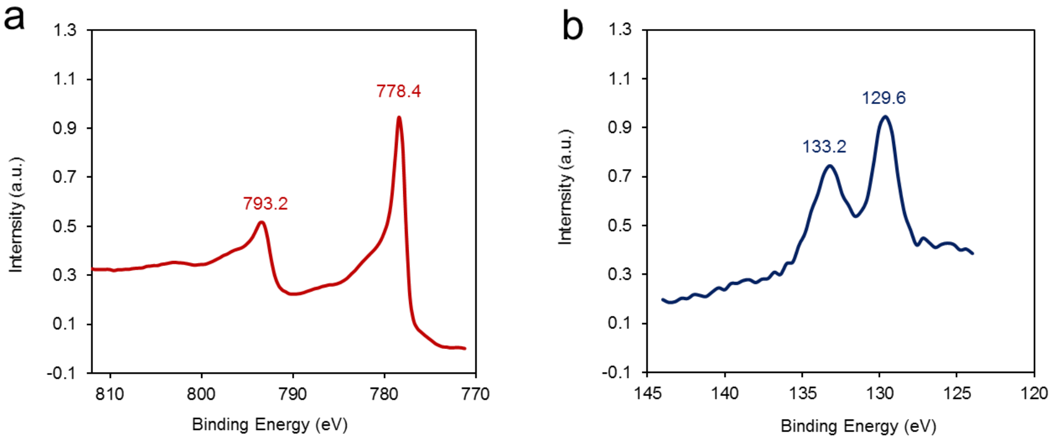

Fig. S4: XPS data of CoP_x. The peak positions of the Co 2p spectra (a) indicate that the Co is primarily metallic-like in oxidation state (Co(0)) while the P 2p spectra show P to be in a mixture of phosphide and phosphate-like forms. The phosphate is likely an oxidized component near the surface.

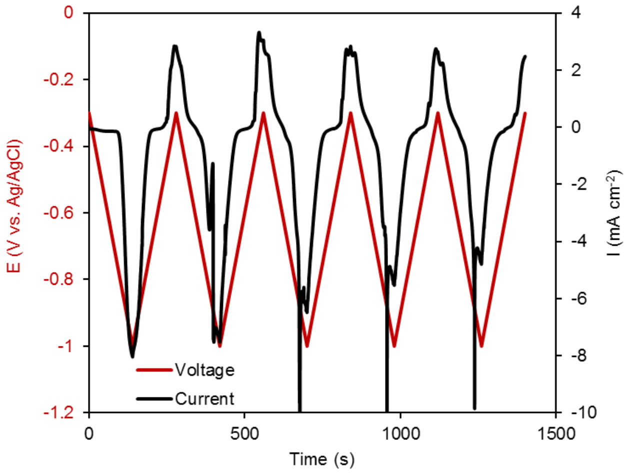

Fig. S5: Current and voltage traces from CoP_x deposition in QCM cell. In the QCM cell, a gold coated quartz electrode was cycled for 5 times under the standard electrodeposition conditions while the current and mass changes were monitored. The total mass and charge passed through the circuit in this series of measurements in illustrated in Fig. 3a in the main text.

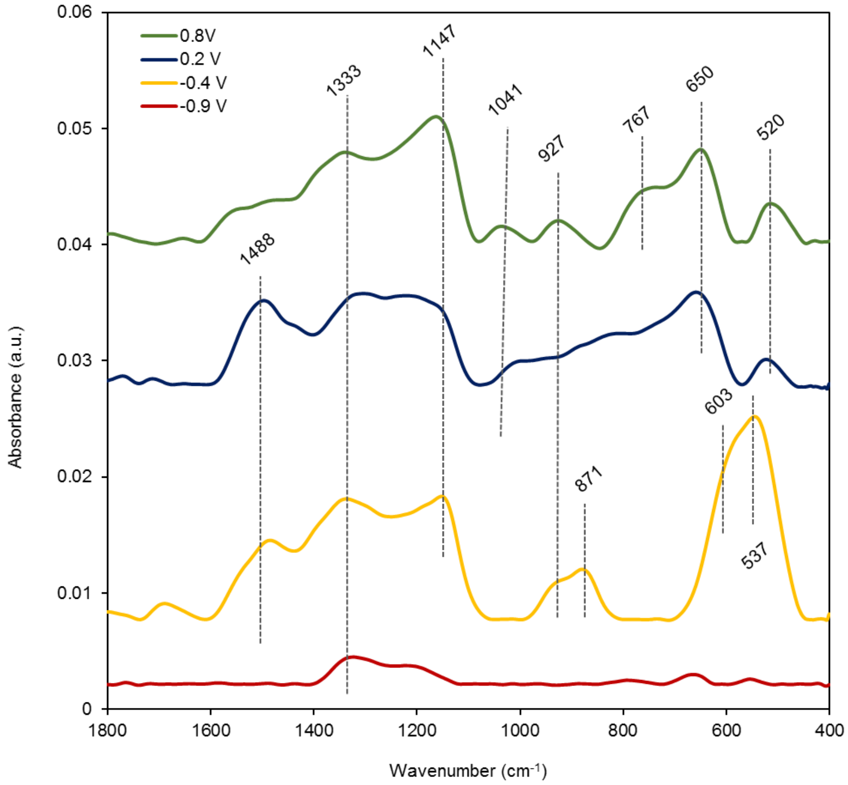

Fig. S6: IR analysis of CoP_x post electrolysis (potentials referenced to NHE). The spectrum is largely featureless at –0.9 V where the cobalt is in a metallic state. Upon increasing the voltage (more positive), new features arise relating to Co-O (~500-650 cm^{-1}), Co-OH(~600-900 cm^{-1}), and phosphate (~1100 cm^{-1}) species.

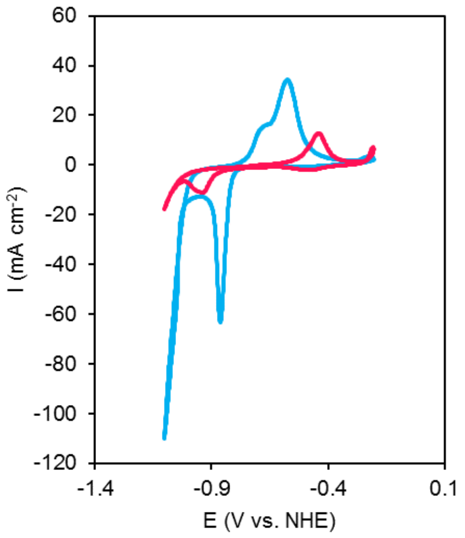

Fig. S7: Inhibition of CoP_x HER current with KSCN. In a CV cycle at 5mV s^{-1} in KOH (1 M), CoP_x (blue) features a dramatically reduced HER current and redox peaks upon the introduction of KSCN (100 mM, magenta). KSCN is a known in inhibitor of metal sites in transition metal catalysts under reducing conditions and the results point to the Co being the active site for the HER.

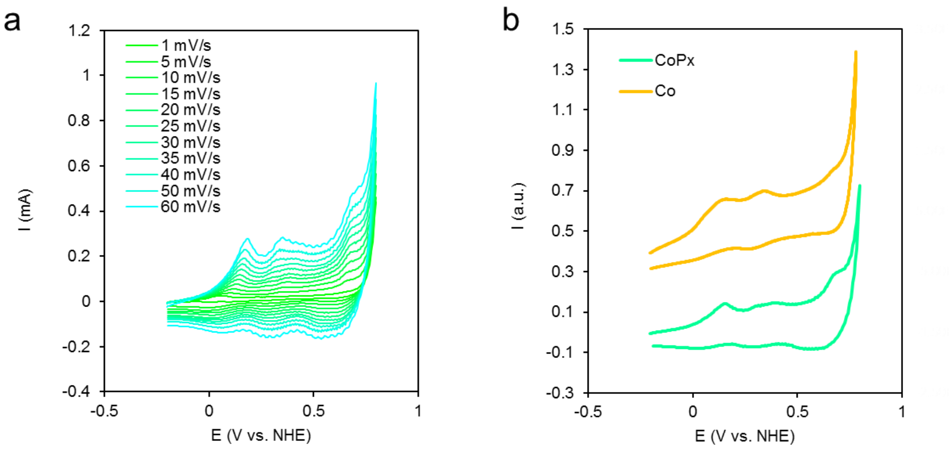

Fig. S8: CV scans in the OER potential window. CoP_x displays 3 sets of redox peaks between 0 and 0.8 V vs. NHE (a). In KOH (1 M) at 30 mV s^{-1}, the CV of CoP_x (turquoise) and electrodeposited Co (yellow) show a similar set of redox peaks before the start of catalytic OER (b). This result points to the likely similarity in surface active phase and active sites for the OER for the two species.

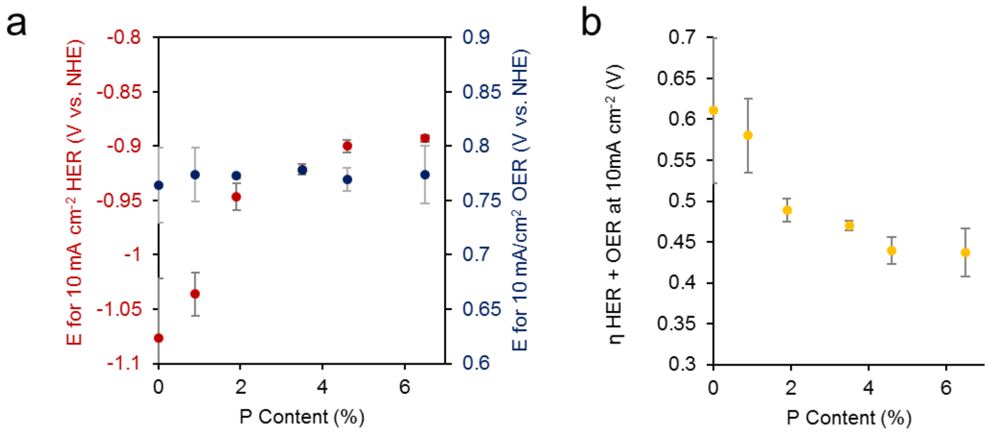

Fig. S9: Effects of P incorporation. CoP_x catalysts were fabricated with an increased P content by systematically increasing the sodium hypophosphite ratio in the deposition solution (0, 30, 100, 500, 1000, and 3000 mM, respectively). The voltage necessary to attain 10 mA cm^{-2} for HER (a) and the overpotential for overall water splitting at 10 mA cm^{-2} was systematically decreased (b).

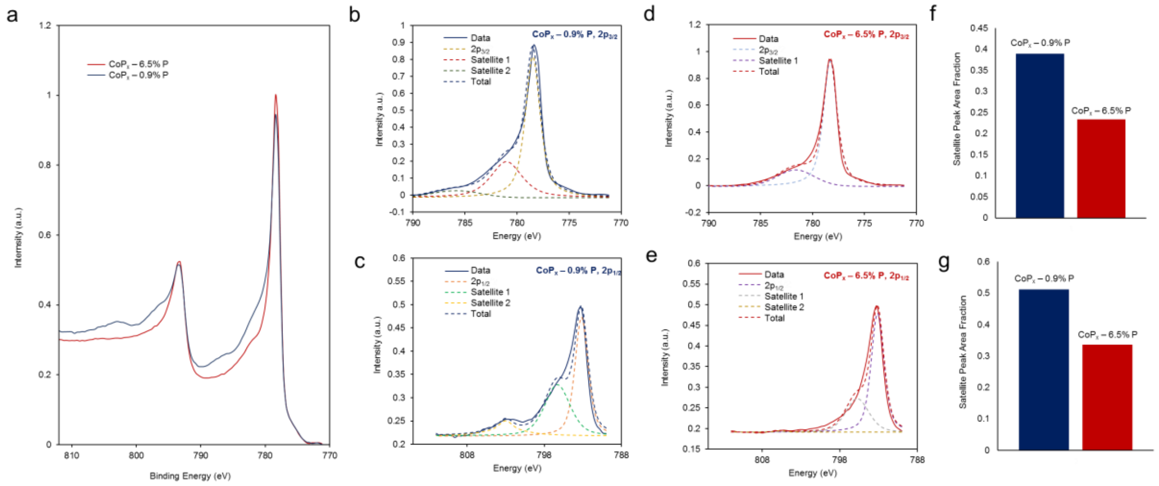

Fig. S10: High resolution XPS analysis. To examine the effects of P incorporation on the electronic structure of Co in CoP_x, we compared the Co 2p XPS peaks of CoP_x with 0.9% and 6.5% P loading, after argon sputtering to remove surface oxides and contamination (a). The Co $2p_{3/2}$ (b,d) and Co $2p_{1/2}$ (c,e) were fit to the main peak ($2p_{3/2}$ or $2p_{1/2}$) and satellite peaks. For both peaks, CoP_x with 0.9% P shows a larger relative area of satellite peaks (f,g), likely stemming from plasmonic losses, presumably indicating a larger amount of valence electrons. However, potential contributions from Co(II) species, which may arise from residual/buried oxides, to the spectra >780 eV cannot be completely discounted.

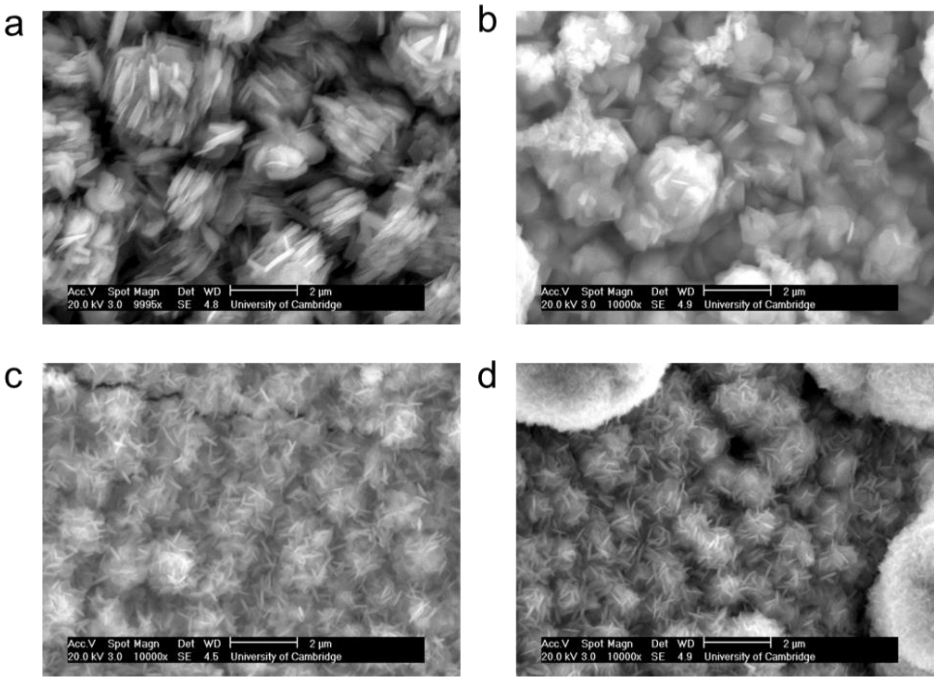

Fig. S11: SEM imaging of $CoFeP_x$ catalysts. Morphologies of $CoFeP_x$ with 6.5% P and 0 (a), 3 (b), 5 (c), and 15 (d) % Fe. The Fe-doped samples were prepared by soaking in an aqueous Fe solution at 80° C for set periods of time. The surface structures of the Fe incorporated films appeared to have smaller plate-like morphologies.

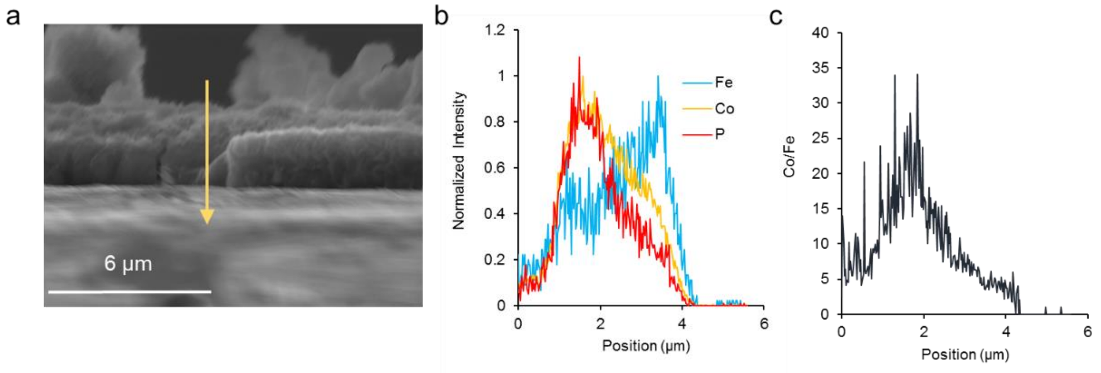

Fig. S12: Elemental analysis of $CoFeP_x$. $CoFeP_x$ elemental composition was probed with an EDS line scan on a thin film cross-section (a). The sample was situated approximately perpendicular to the electron beam. Normalized intensities of Co, Fe, and P reveal that the surface is Fe-rich (b). Plotting the Co/Fe ratio illustrates a surface ratio of ~4:1 and a bulk ratio of ~10-20:1 (c). Because the Fe diffuses from the solution through the surface, the surface of $CoFeP_x$ is naturally enriched with Fe.

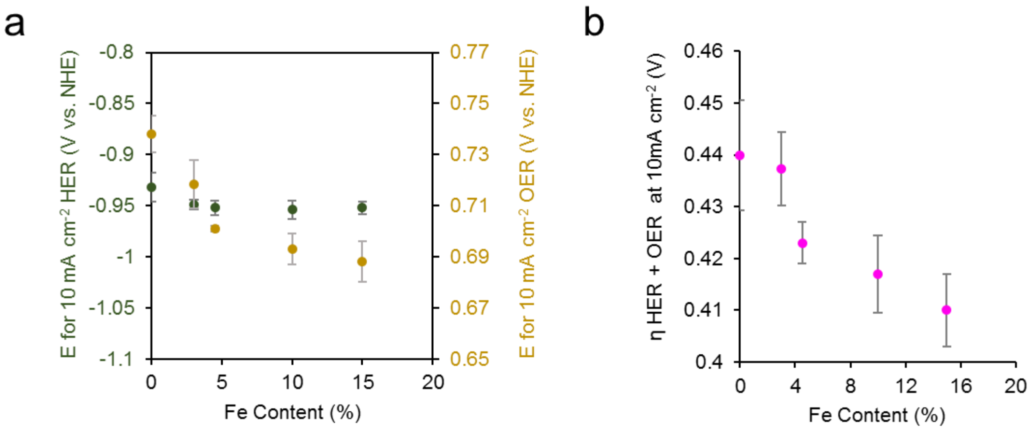

Fig. S13: Effects of Fe doping. $CoFeP_x$ catalysts were fabricated by immersing CoP_x with 6.5% P into a 100 mM solution of $FeCl_2$ at 80° C for durations of 0, 0.5, 5, 15, and 60 min. The OER activity, measured as the overpotential necessary to apply to attain a working current density of 10 mA cm^{-2} increased (a), which led to a decrease in the overpotential for overall water splitting at 10 mA cm^{-2} (b). The HER activity was relatively unchanged so the improvement for overall water electrolysis was due to the beneficial effects of the Fe dopants on the OER catalysis.

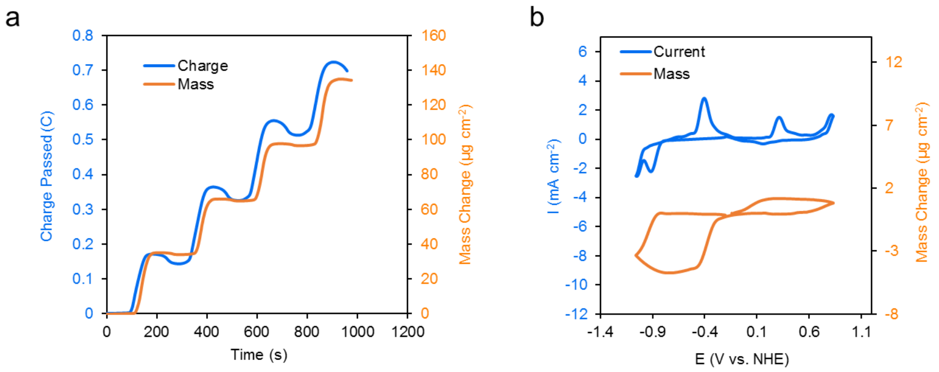

Fig. S14: QCM probing of $CoFeP_x$ catalyst. The $CoFeP_x$ deposition and catalytic cycle was probed with the QCM and showed a similar trend in step-by-step layer buildup during the deposition (a). Mass changes corresponding to redox peaks were also observed (b), which were very similar to the CoP_x catalyst. This indicates that the $CoFeP_x$ also reduces away its oxide shell prior to HER catalysis and undergoes transitions through to various higher-valent oxide phases on its surface prior to OER catalysis.

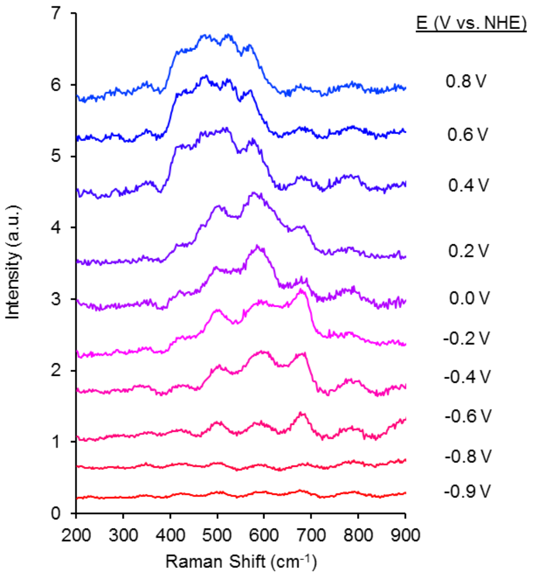

Fig. S15: *Operando* Raman probing of $CoFeP_x$. In a similar fashion to CoP_x, the Raman-active modes were probed for $CoFeP_x$ and revealed a potential dependent phase interconversion between metallic, spinel, and layered double hydroxide structures. Using a 473 nm diode laser and the quartz crystal microbalance spectroelectrochemical cell (1.0 M KOH electrolyte solution), the $CoFeP_x$ metastable sirface phases and transient behavior is captured.

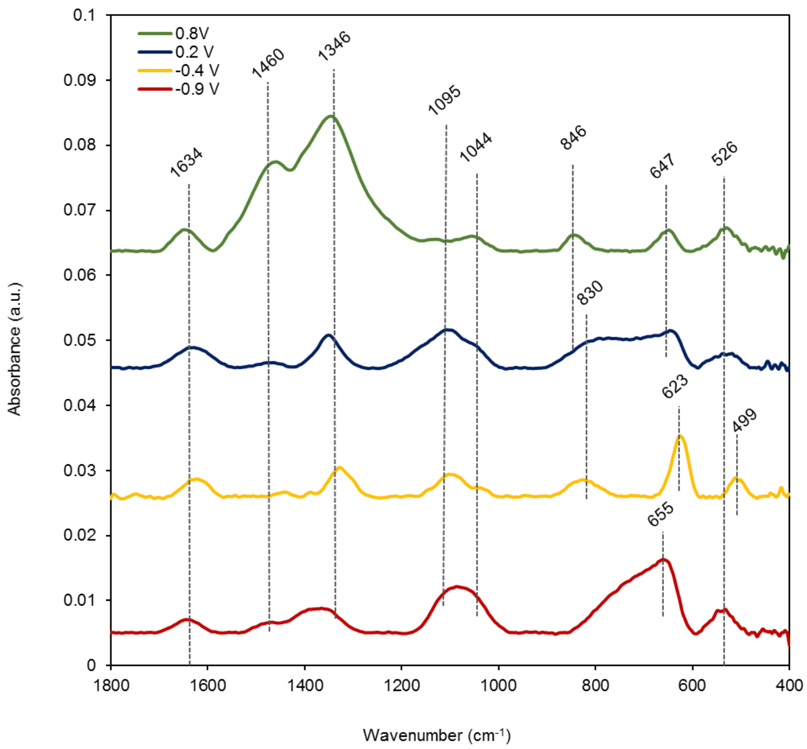

Fig. S16: $CoFeP_x$ post electrolysis Infrared spectra. Spectral evolution in the 400-900 cm^{-1} range points to surface phase interconversion between spinel and layered double hydroxide morphologies as potentials shift more positive (referenced to NHE). The presence of surface phosphates at 1000-1150 cm^{-1} is also evident. Peaks around 1300-1400 could potentially stem from an intercalated species in the layered structure, potentially acetate or carbonate. Like in the Raman spectra, differences between CoP_x and $CoFeP_x$ are evident in terms of peak position and relative intensities, especially in the M-O and M-OH regions (~500-650 and ~650-900 cm^{-1}).

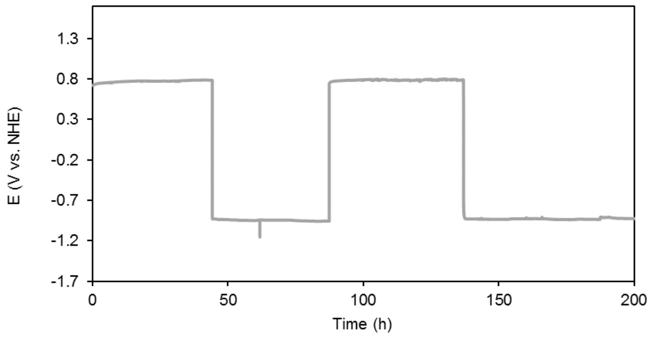

Fig. S17: $CoFeP_x$ stability testing. $CoFeP_x$ stability was probed through chronopotentiometric testing at 10 and –10 mA cm^{-2}, with periodic switching between the two in aqueous KOH (1 M). This was tested in a three-electrode configuration with a Ag/AgCl reference electrode and glassy carbon counter electrode. The potential of the reference was checked before and after the measurement to exclude effects of potential drift on the stability data.

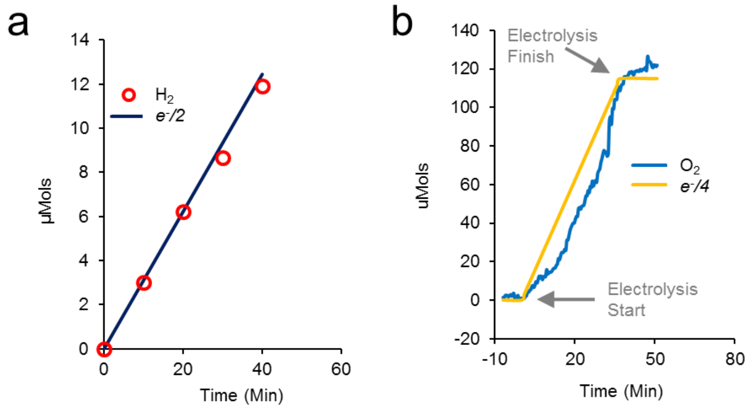

Fig. S18: Product quantification. Hydrogen (a) was measured by gas chromatography, using methane as an internal standard. Oxygen (b) was measured with an Ocean Optics Neofox Fospor fluorescent sensor. Faradaic yields were nearly quantitative for both products, indicating that the current measured stems from catalytic HER and OER reactions. The experiments were carried out with a three-electrode configuration in 1.0 M KOH electrolyte solution with a Ag/AgCl reference electrode and a glassy carbon working electrode.

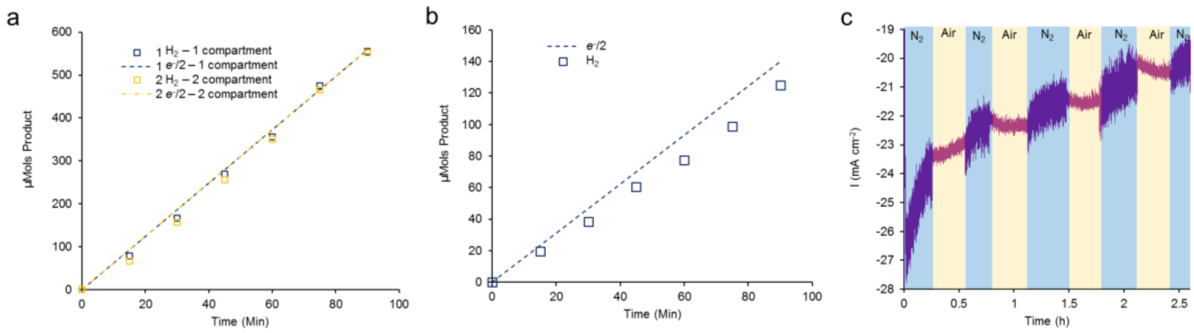

Fig. S19: H_2 quantification in the absence and presence of O_2. The Faradaic yield for H_2 evolution was ~99% when testing in a 2 compartment that was O_2-free or a 1 compartment setup where O_2 was first removed by N_2 purging but then generated at the counter electrode to produce low O_2 concentrations (a). Under an air environment, the Faradaic yield decreased to ~90% (b). During chronoamerometric testing at 0.9 V vs. NHE, a slight increase in current density was observed when switching from nitrogen to air environments (c), indicating that partial reduction of O_2 takes place, likely leading to the decrease in Faradaic yields for H_2.

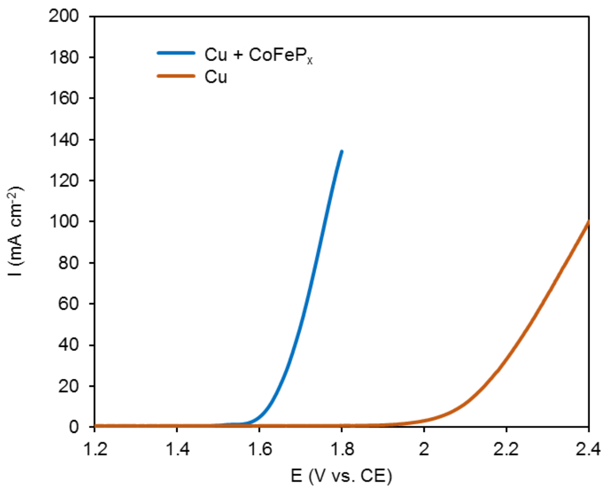

Fig. S20: Control CV with bare Cu substrate. The copper substrate was tested in a two-electrode setup for overall water electrolysis and compared with the $CoFeP_x$ deposited on copper. The copper foil showed negligible currents at the range tested, indicating that there is little potential contribution to the catalytic currents within the voltage range of interest. Experiments were performed at 25°C in 1.0M KOH electrolyte solution with a two electrode configuration.

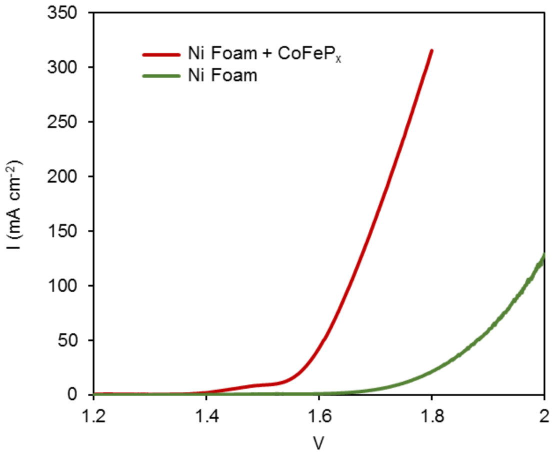

Fig. S21: Control CV with bare Ni foam substrate. Nickel Foil was also tested and compared to the $CoFeP_x$ on nickel foil. The $CoFeP_x$ catalyst outperformed the Nickel foil by 300-400 mV. Experiments were performed at 25°C in 1.0M KOH electrolyte solution with a two electrode configuration.

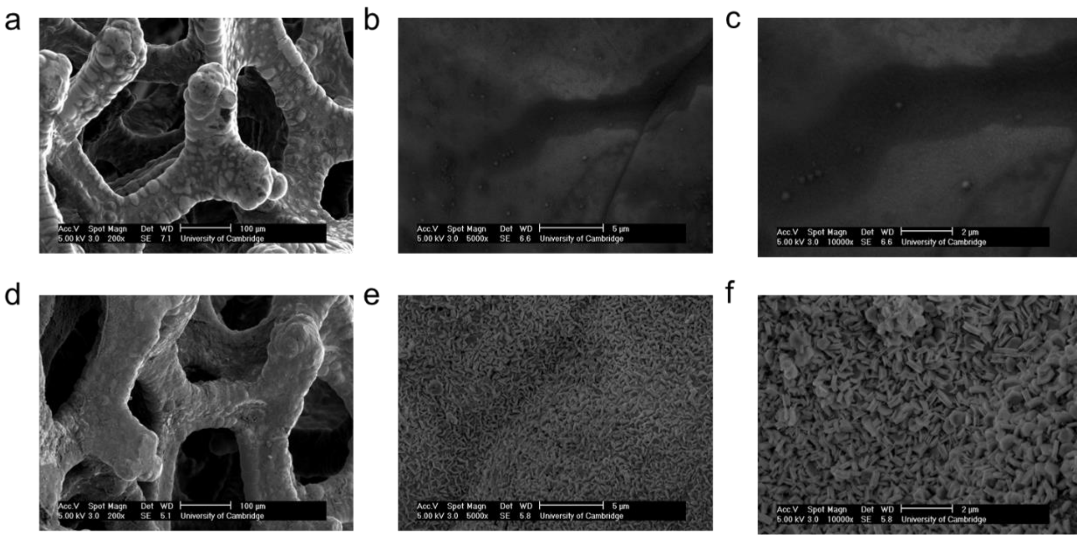

Fig. S22: SEM images of $CoFeP_x$/Ni foam. Nickel foam substrates featured a porous network of ~100 µm fibers with flat surfaces (a-c). A high surface area substrate would allow for increased catalyst loading and enhanced current densities per projected surface area. $CoFeP_x$ uniformly coated the nickel foam substrates and had a flake-like morphology (d-f). The morphology on the $CoFeP_x$ on the nickel foam substrates was similar to that of $CoFeP_x$ deposited on a flat copper substrate.

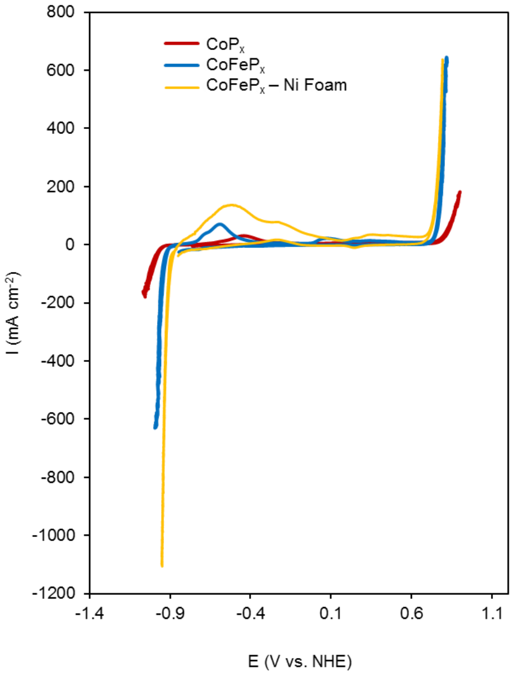

Fig. S23: Characterization of $CoFeP_x$ on Ni foam. The $CoFeP_x$ performance was evaluated with cyclic voltammetry in a three electrode configuration and KOH (1.0 M) electrolyte solution and compared to the same material deposited on a planar copper foil. A decrease in overpotential for each half reaction is attained through an increase in surface area and consequent larger active site density per projected surface area.

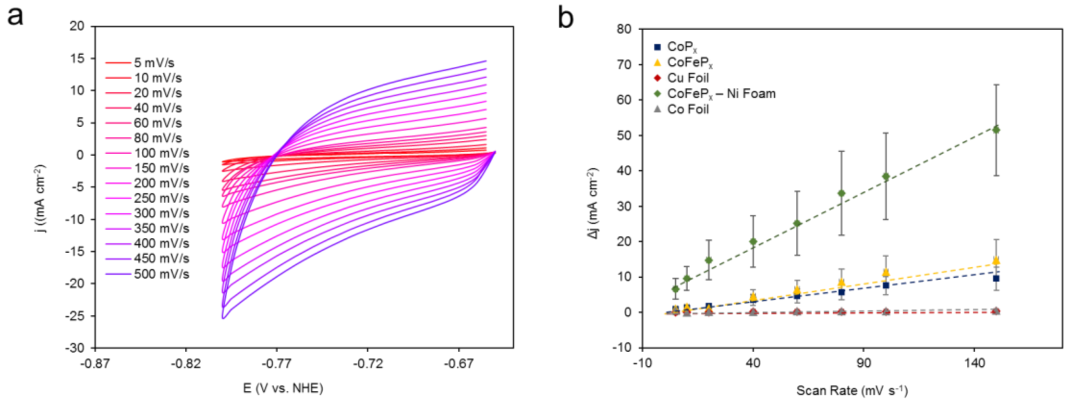

Fig. S24: Double-layer capacitance measurements. Increases in surface area of CoP_x and $CoFeP_x$ on planar and Ni-foam substrates were estimated by measuring the capacitive current as a function of scan rate (a). From the slope of current vs. scan rate, roughness factor of $CoFeP_x$ and $CoFeP_x$-Ni foam were approximated to be 24 and 84, respectively. The similarity in CoP_x and $CoFeP_x$ estimated surface areas signifies that the decrease in overpotential for water electrolysis is due to differences in the catalytic activity of the active site rather than from an increased surface area.

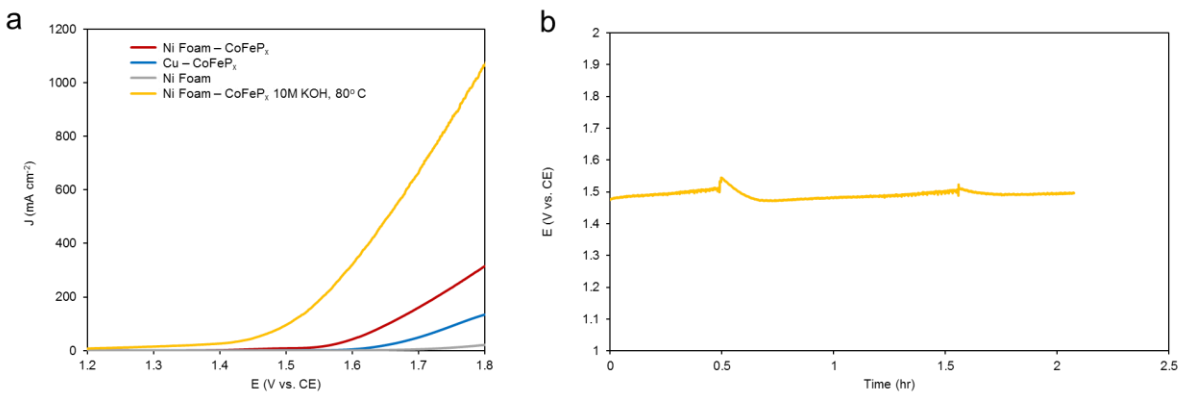

Fig. S25: Water electrolysis under harsh conditions. In a 10M KOH electrolyte at 80° C, $CoFeP_x$ on Ni foam can perform overall water electrolysis at currents of ~100 mA cm^{-2} at 1.5 V total voltage (a). This performance does not decrease within the timescale of a 2 hour chronopotentiometric measurement (b).

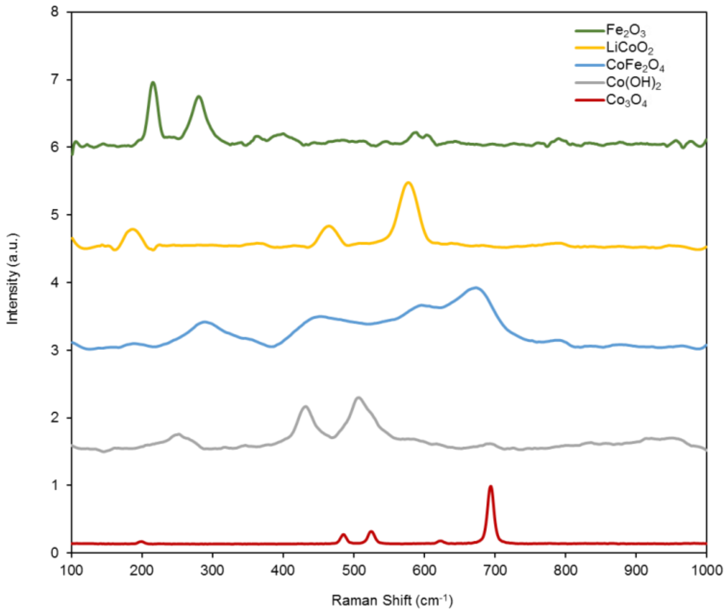

Fig. S26: Raman spectra of commercially available standards. Standard spectra were used to compare to *operando* Raman measurements of CoP_x and $CoFeP_x$. The spectra of the standards were acquired under the same conditions as those for the CoP_x and $CoFeP_x$ spectra (473 nm diode laser).

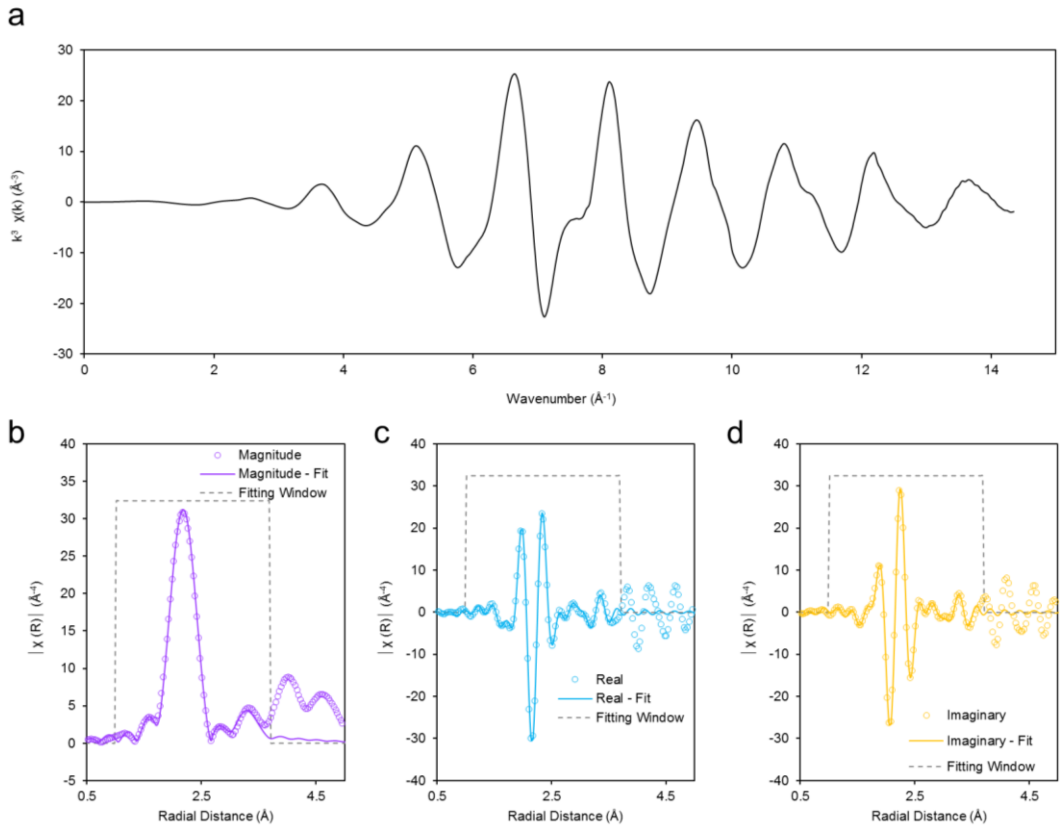

Fig. S27: EXAFS data and fitting. Raw data in k-space (a), EXAFS spectra of CoP_x and fits of the magnitude (b), real component (c) and imaginary components (d) of the spectra. The data is used to visualize the immediate chemical environment around the Co in the CoP_x catalyst.

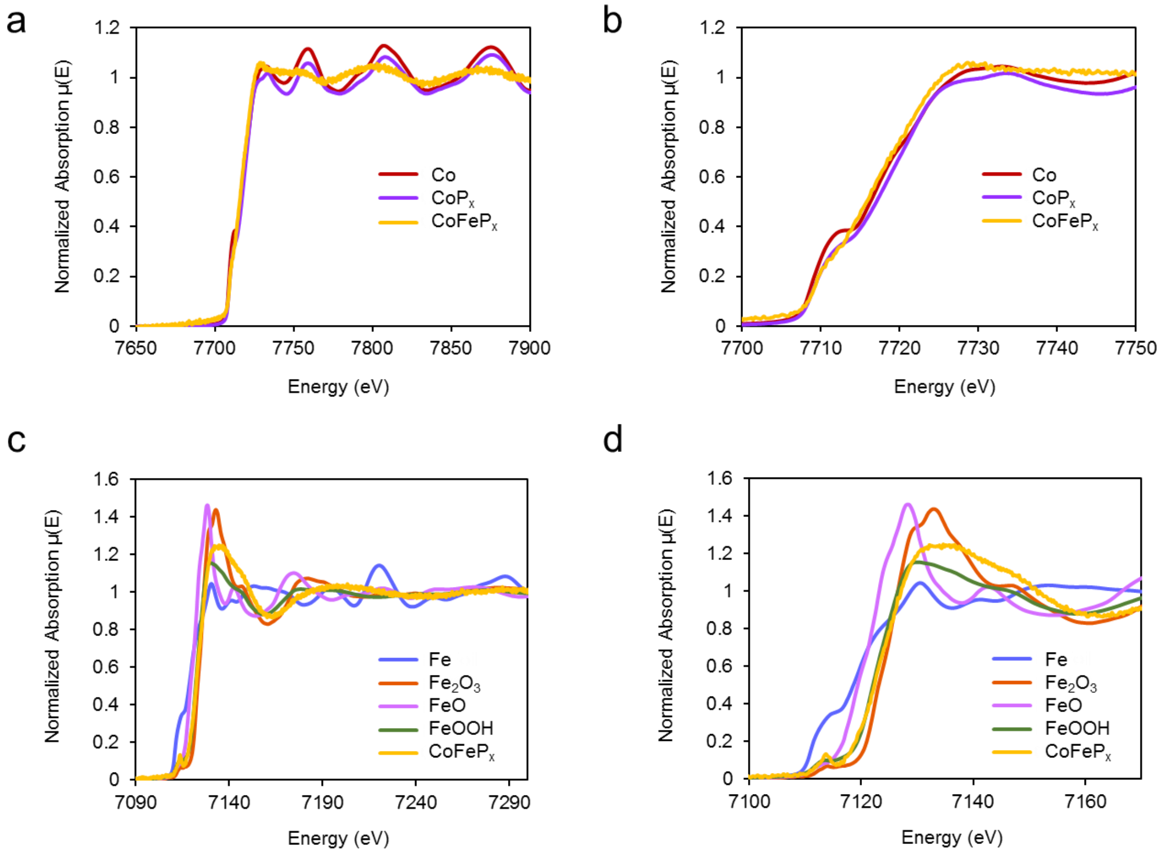

Fig. S28: *Ex situ* X-ray absorption near-edge spectroscopy (XANES) characterization of $CoFeP_x$. The cobalt K-edge spectrum of $CoFeP_x$ (a,b) indicates that the Co is primarily in a metallic state. Likewise, the Fe K-edge spectra of $CoFeP_x$ (c,d), indicate that the Fe is in an oxidation state and environment similar to FeOOH and Fe_2O_3, judging from the spectral similarity of $CoFeP_x$ to the aforementioned standards.

References

1. N. Jiang, B. You, M. Sheng and Y. Sun, *Angew. Chem., Int. Ed.*, 2015, **54**, 6252-6254.
2. H. Wang, H.-W. Lee, Y. Deng, Z. Lu, P.-C. Hsu, Y. Liu, D. Lin and Y. Cui, *Nat. Commun.*, 2015, **6,** 7261.
3. H. Li, S. Chen, X. Jia, B. Xu, H. Lin, H. Yang, L. Song and X. Wang, *Nat. Commun.*, 2017, **8,** 15377.
4. M. Ledendecker, S. Krick Calderón, C. Papp, H-P. Steinrück, M. Antonietti and M. Shalom, *Angew. Chem., Int. Ed.*, 2015, **54**, 12361-12365.
5. L.-A. Stern, L. Feng, F. Song and X. Hu, *Energy Environ. Sci.*, 2015, **8**, 2347-2351.
6. Y. Jin, H. Wang, J. Li, X. Yue, Y. Han, P. K. Shen and Y. Cui, *Adv. Mater.*, 2016, **28**, 3785-3790.
7. J. Zhang, T. Wang, D. Pohl, B. Rellinghaus, R. Dong, S. Liu, X. Zhuang and X. Feng, *Angew. Chem., Int. Ed.*, 2016, **55**, 6702-6707.
8. J. Luo, J.-H. Im, M. T. Mayer, M. Schreier, M. K. Nazeeruddin, N.-G. Park, S. D. Tilley, H. J. Fan and M. Grätzel, *Science*, 2014, **345**, 1593-1596.
9. C. Tang, N. Cheng, Z. Pu, W. Xing and X. Sun, *Angew. Chem., Int. Ed.*, 2015, **54**, 9351-9355.
10. Y. Hou, M. R. Lohe, J. Zhang, S. Liu, X. Zhuang and X. Feng, *Energy Environ. Sci.*, 2016, **9**, 478-483.
11. J. Tian, N. Cheng, Q. Liu, X. Sun, Y. He and A. M. Asiri, *J. Mater. Chem. A*, 2015, **3**, 20056-20059.
12. X. Gao, H. Zhang, Q. Li, X. Yu, Z. Hong, X. Zhang, C. Liang and Z. Lin, *Angew. Chem., Int. Ed.*, 2016, **55**, 6290-6294.
13. G. F. Chen, T. Y. Ma, Z. Q. Liu, N. Li, Y. Z. Su, K. Davey and S. Z. Qiao, *Adv. Funct. Mater.*, 2016, **26**, 3314-3323.
14. L. Jiao, Y.-X. Zhou and H.-L. Jiang, *Chem. Sci.*, 2016, **7**, 1690-1695.
15. W. Zhu, X. Yue, W. Zhang, S. Yu, Y. Zhang, J. Wang and J. Wang, *Chem. Comm.*, 2016, **52**, 1486-1489.
16. B. You, N. Jiang, M. Sheng, M. W. Bhushan and Y. Sun, *ACS Catal.*, 2015, **6**, 714-721.
17. Y. Wu, G. D. Li, Y. Liu, L. Yang, X. Lian, T. Asefa and X. Zou, *Adv. Funct. Mater.*, 2016, **26**, 4839-4847.
18. X. Wang, W. Li, D. Xiong, D. Y. Petrovykh and L. Liu, *Adv. Funct. Mater.*, 2016, **26**, 4067-4077.
19. Z. Pei, L. Xu and W. Xu, *Appl. Surf. Sci.*, 2018, **433**, 256-263.
20. X. Wang, W. Li, D. Xiong and L. Liu, *J. Mater. Chem. A*, 2016, **4**, 5639-5646.
21. L. Yu, H. Zhou, J. Sun, F. Qin, F. Yu, J. Bao, Y. Yu, S. Chen and Z. Ren, *Energy Environ. Sci.*, 2017, **10**, 1820-1827.
22. H. Shi, H. Liang, F. Ming and Z. Wang, *Angew. Chem., Int. Ed.*, 2017, **56**, 573-577.
23. Y. Wang, C. Xie, D. Liu, X. Huang, J. Huo and S. Wang, *ACS Appl. Mater. Interfaces*, 2016, **8**, 18652–18657.
24. Z. Zhao, D. E. Schipper, A. P. Leitner, H. Thirumalai, J.-H. Chen, L. Xie, F. Qin, M. K. Alam, L. C. Grabow S. Chen, D. Wang, Z. Ren, Z. Wang, K. H. Whitmire, and J. Bao, *Nano Energy*, 2017, **39**, 444-453.
25. Y. Zhu, W. Zhou, Y. Zhong, Y. Bu, X. Chen, Q. Zhong, M. Liu and Z. Shao, *Adv. Energy Mater.*, 2017, **7**, 160212.
26. J. Hou, Y. Sun, Y. Wu, S. Cao and L. Sun, *Adv. Funct. Mater.*, 2018, **28**, 1704447.
27. J. Li, J. Li, X. Zhou, Z. Xia, W. Gao, Y. Ma and Y. Qu, *ACS Appl. Mater. Interfaces*, 2016, **8**, 10826-10834.

End of Electronic Supporting Information
